# Effect of Impurities on the Formation of End-Group Clusters in Natural Rubber: Phenylalanine Dipeptide as an Impurity Protein

**DOI:** 10.1021/acs.macromol.3c01833

**Published:** 2024-03-05

**Authors:** Mayank Dixit, Takashi Taniguchi

**Affiliations:** Graduate School of Engineering, 74062 Kyoto University, Nishikyo-ku, Kyoto 615-8510, Japan

## Abstract

Natural rubber (NR), containing nonrubber constituents such as proteins, exhibits exceptional characteristics including high toughness, tear resistance, and wet skid resistance. Gaining a thorough understanding of the interplay between proteins and the terminal groups of the *cis*-1,4-polyisoprene chains in NR is vital for comprehending the superior properties of NR in comparison to synthetic polyisoprene rubber. The terminal ends of the *cis*-1,4-polyisoprene chains in NR encompass two distinct types of terminal groups: ω terminals and α terminals. Extensive investigations employing solid-state NMR analysis have revealed the structures of the ω and α terminals in NR, identifying ω as dimethyl allyl-(*trans*-1,4-isoprene)_2_ (DMA), while the α terminals have been categorized into six types [Oouchi, M.; Ukawa, J.; Ishii, Y.; Maeda, H. Structural analysis of the terminal groups in Commercial Hevea Natural Rubber by 2D-NMR with DOSY filters and multiple-WET methods using ultrahigh-field NMR. *Biomacromolecules* **2019,** *20,* 1394–1400]. In this study, our primary focus is to explore the interaction between phenylalanine dipeptide (PAP) and the terminal groups within six types of melt systems (_ω_PI_α*n*_ + P, *n* = 1···6, “P” stands for PAP). By utilizing equilibrated systems, various physical quantities were estimated, including end-to-end distance (*R*_ee_), radius of gyration (*R*_g_), end-to-end vector autocorrelation function (*C*(*t*)), average rotational relaxation time τ_rot_, self-diffusion coefficients of polymer chains, radial distribution functions (RDFs) of terminal groups around PAP, and the survival probability *P*(τ) for terminal groups surrounding PAP. Analysis of *C*(*t*) and τ_rot_ unveiled that PAP significantly hinders the dynamics of hydroxy-terminated and ester-terminated polyisoprene chains in the _ω_PI_α1‑to‑α6_ melt systems. Examination of RDFs demonstrated a robust association between PAP molecules and α terminals compared to ω terminal groups. Moreover, the local density of α terminal groups around other α terminal groups was notably reduced in the presence of PAP. The association between PAP and DMA was found to be weaker than that of DMA and DMA, indicating a weak correlation between PAP molecules and ω terminal groups. By employing the potentials of mean force, we conducted an investigation to calculate the cluster formation fraction of terminal groups associated with PAP as well as terminal groups forming clusters of various sizes in the 13 melt systems. Our findings revealed that in the _H_PI_H_, _ω_PI_α1_, _ω_PI_α3_, and _ω_PI_α5_ systems, firm cluster formation was not observed without PAP. However, in the presence of PAP, stable clusters comprising PAP – α1, PAP – α3, and PAP – α5 were formed. Conversely, in the _ω_PI_α2_ + P, _ω_PI_α4_ + P, and _ω_PI_α6_ + P systems, stable clusters involving α2 and α2, PAP and α2, α4 and α4, PAP and α4, α6 and α6, and PAP and α6 with sizes ranging from 2 to 9 were observed. These findings provide evidence for the formation of physical junction points (PJPs) between PAP molecules and hydroxy- or ester-terminated polyisoprene chains through their respective α1, α2, α3, α4, α5, and α6 terminals. Notably, the formation of globular PJPs between PAP and ester terminal groups was observed, while networked PJPs were established between PAP molecules and hydroxy terminal groups. These PJPs are postulated to be responsible for the superior comprehensive properties exhibited by NR in comparison to synthetic polyisoprene.

## Graphical abstract

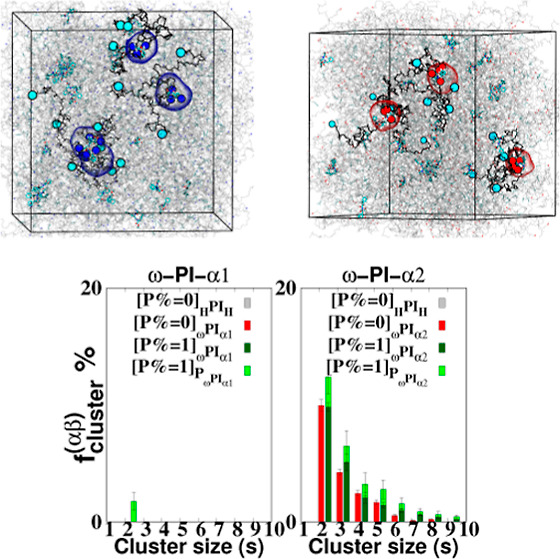

## 1 Introduction

The composition of natural rubber (NR), obtained from the *Hevea brasiliensis* plant, comprises approximately 94 wt % *cis*-1,4-polyisoprene and 6 wt % nonrubber components, including proteins, phospholipids, fatty acids, and carbohydrates.^2–14^ The outstanding properties exhibited by NR, such as elasticity, abrasion resistance, resilience, impact resistance, efficient heat dispersion, and malleability at low temperatures, are not solely attributed to the cis content, stereoregularity, and high molecular weight of polyisoprene but also closely tied to the structure of the terminal end groups of the polyisoprene chains within NR.^15–17^ These unique properties of NR cannot be fully replicated in synthetic polyisoprene rubber (IR),^16,18^ where the main chain structure is similar to that of NR, but properties such as tensile strength and strain-induced crystallization significantly lag behind those of NR.^18^ Consequently, NR finds extensive utilization in various applications, including the manufacturing of tires for aircraft and heavy trucks.

The polar terminal ends of NR play a significant role in its mechanical performance.^19^ The polymer chains in NR consist of *cis*-1,4-polyisoprene with ω-terminals and α-terminals. It has been reported that the ω-terminals contain a dimethylallyl group (DMA) and two to three trans isoprene units,^1,17,20^ while the α-terminals are considered to consist of hydroxy, ester,^1,11,17,20–22^ and phosphate groups.^8^ The resistance of rubber to deformation and fracture before vulcanization, known as green strength, has also been explored.^23^ Current understanding suggests that nonrubber components, particularly proteins and phospholipids, form distinct natural network structures with the terminal groups of *cis*-1,4-polyisoprene, which are crucial for the excellent mechanical properties of NR, including high toughness, tensile strength, and crack growth resistance.^15–17,24–29^ The association of proteins with ω-terminals and phospholipids with α-terminals, leading to the formation of branch points in the polymer, has been speculated.^8^ These branch points play a vital role in polymer chain elongation during deformation. Extensive investigations of these functional groups using NMR by Tanaka et al.^8–10^ have led to the presumption that proteins form associations with ω-terminal groups, while phospholipid molecules associate with α-terminal groups through either hydrogen bonding or ionic bonding.^8–10^ Recent experimental studies have indicated that the formation of physical junction points (PJPs) between the terminal groups of polyisoprene chains in NR is crucial for strain-induced crystallization.^4,13,30–32^ Tanaka et al.^17^ have reported the presence of phenylalanine di- and tripeptides and proposed that proteins or oligopeptides are associated with ω-terminals, while phospholipids are linked with α-terminals. Other studies have also suggested that ω-terminals are bonded with oligopeptides or proteins, while α-terminals are bonded with lipid molecules.^8,33^ Conversely, Wu et al.^5^ have reported that proteins are bonded with α-terminals, while lipids are bonded with ω-terminals using stochastic optical reconstruction microscopy (STORM). However, the structural mechanism underlying the formation of PJPs between peptides or proteins and terminal groups remains elusive.

In this study, we aim to elucidate the association of phenylalanine dipeptide (PAP) with ω-terminals and/or α-terminals as well as investigate whether stable PJPs can be formed between PAP and the terminal groups of polyisoprene chains. To achieve this, all-atom molecular dynamics (MD) simulations were performed. It is important to note that our investigation is based on a specific chemical model for *cis*-1,4-polyisoprene that mimics the structure found in the NR tree. The chemical structures of the ω and α terminals in our molecular model for polyisoprene are based on recent experimental findings by Ohuchi et al.^1^ where α terminal groups were classified into six, and further into two groups, odd numbered and even numbered alpha terminals, i.e., α_(2*m*–1)_ contains an ester group, and α_(2*m*)_ contains a hydroxy-group (*m* = 1, 2, 3). Molecular modeling and MD simulations have played crucial roles in polymer research, providing molecular-level insights into the macroscopic mechanical and chemical properties of polymer materials that are challenging to obtain through experimental studies.^34–60^ In our recent study,^22,61,62^ we employed a novel multiscale modeling approach to investigate the association behavior of α-terminals of hydroxy-terminated and ester-terminated *cis*-1,4-polyisoprene chains, utilizing a technique developed by Choi et al.^63^ In the present work, we consider *cis*-1,4-polyisoprene with ω-terminals and α_*n*_-terminals^1^ (where *n* ranges from 1 to 6) in the presence and absence of PAP. We examine 13 different types of melt systems with distinct ω-terminals and α-terminals, utilizing the same multiscale methodology employed in our previous work.^22,61,62^ Detailed information on each melt system can be found in Table 1, and the extended structures of the different types of polyisoprene chains are depicted in Figure S1.

**Table 1:** Details of the Melt Systems Employed in the Study^a^

code	systems	*cis*-isoprene	terminal 1	terminal 2	*N*	*M*	*P* (wt %)	*NVT* (ns)	sim. annl. (ns)	*NPT* (ns)	*NVT* production run (ns)
PI_0_	_H_PI_H_	24	isoprene	isoprene	24	512	0	20	20	100	1000
PI_I_	_ω_PI_α1_	21	ω	α1	24	512	0	20	20	100	1000
PI_II_	_ω_PI_α2_	21	ω	α2	24	512	0	20	20	100	1000
PI_III_	_ω_PI_α3_	21	ω	α3	24	512	0	20	20	100	1000
PI_IV_	_ω_PI_α4_	21	ω	α4	24	512	0	20	20	100	1000
PI_V_	_ω_PI_α5_	21	ω	α5	24	512	0	20	20	100	1000
PI_VI_	_ω_PI_α6_	21	ω	α6	24	512	0	20	20	100	1000
PI_I_ + P	_ω_PI_α1_ + P	21	ω	α1	24	512	1	100	20	200	1000
PI_II_ + P	_ω_PI_α2_ + P	21	ω	α2	24	512	1	100	20	200	1000
PI_III_ + P	_ω_PI_α3_ + P	21	ω	α3	24	512	1	100	20	200	1000
PI_IV_ + P	_ω_PI_α4_ + P	21	ω	α4	24	512	1	100	20	200	1000
PI_V_ + P	_ω_PI_α5_ + P	21	ω	α5	24	512	1	100	20	200	1000
PI_VI_ + P	_ω_PI_α6_ + P	21	ω	α6	24	512	1	100	20	200	1000

a The parameter *N* denotes the count of monomers comprising each polymer chain, while *M* signifies the number of chains contained within each melt system. Additionally, *P* represents the weight percentage of PAP present in each melt system. To perform comprehensive analysis, the full-atom MD simulations were meticulously conducted following a predefined sequence, namely, *NVT* equilibration, simulated annealing, *NPT* equilibration, and *NVT* production run.

## 2 Methods and Computational Details

In this study, we conducted an examination of 13 distinct types of *cis*-1,4-polyisoprene melt systems, each characterized by different combinations of ω-terminal and α-terminal groups, with and without the presence of PAP (as outlined in Table 1). The chemical structure of PAP is depicted in Figure 1. To ensure a comprehensive analysis, we considered polymer melt systems composed of *M* polymer chains, with each chain consisting of *N* monomers (where *M* = 512 and *N* = 24). This choice of combination and size corresponds to a similar approach adopted in our previous work.^22,61,62^ Experimental investigations have revealed that NRs with lower molecular weights exhibit enhanced mechanical and rheological properties, primarily due to a higher proportion of short chains.^64–66^ It is believed that the terminal groups of these short chains readily associate and interact with nonrubber components such as proteins, lipids, and sugars, thereby facilitating the formation of PJPs.^64^ Conversely, NRs with longer molecular chains exhibit inferior mechanical and physical properties as the lower density of chain end groups leads to reduced junction point formation. Therefore, in order to gain insights into the molecular-level behavior of terminal group aggregates, we focused on short polyisoprene chains in our investigation. The molecular weight range of natural polyisoprene extracted from rubber plants spans from 100 to 2000 kDa,^67,68^ which is considerably large and impractical to simulate directly in our numerical simulations. Hence, our study aimed to investigate the specific role of end groups in the formation of polar and nonpolar PJPs in conjunction with PAP. To achieve this objective, we selected a *cis*-1,4-polyisoprene (PI) chain with a molecular weight of 1.6 kDa, which offers a suitable length for the purposes of our investigation.

**Figure 1:**
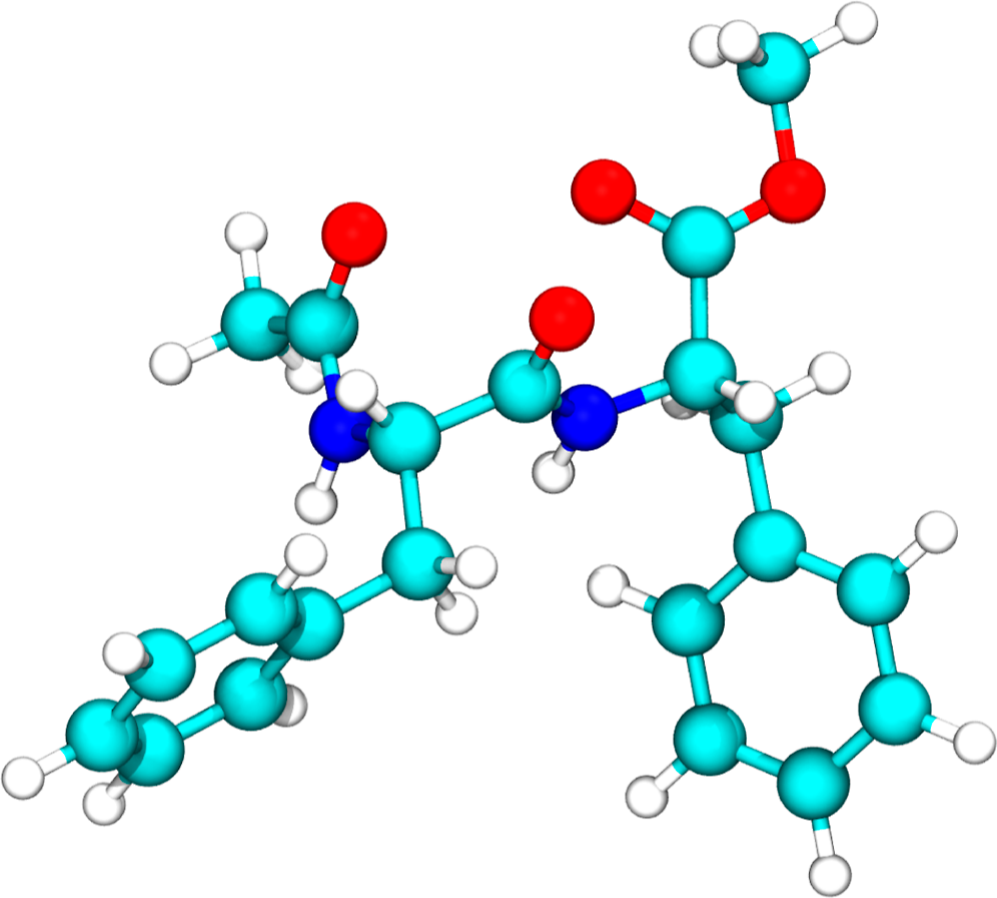
Structure of PAP. The carbon, hydrogen, oxygen and nitrogen atoms are shown by cyan, white, red, blue colors, respectively.

### 2.1 All-Atom MD Simulation Details

The initial configurations of each melt system, excluding PAP, were generated using a multiscale approach, as outlined in our previous work.^22,61,62^ The PAP molecules are randomly distributed in each melt system by packmol software^69^ and multicomponent assembler of CHARMMM-GUI.^70^ These initial configurations were subsequently employed for all-atom MD simulations using GROMACS (version—2019.2).^71^ To generate the necessary input files compatible with GROMACS for each melt system, CHARMM-GUI was employed.^72–74^ The CHARMM general all-atom force field,^75,76^ which has been extensively validated for various polymer melt systems,^63^ was employed for the all-atom MD simulations. The temperature and pressure were set to 360 K and 1 bar, respectively, mirroring the conditions used in our prior research.^22,61,62^ In order to attain equilibrium states for the listed systems­(PI_0‑to‑VI_ and PI_I‑to‑VI_ + P) outlined in Table 1, the initial configurations underwent an energy minimization process. Subsequently, the energy-minimized structures were employed to perform *NVT* equilibration, *NPT* simulated annealing, and *NPT* equilibrations (the simulation times for each step are presented in Table 1). The resulting equilibrated structures (as depicted in Figure S2) were then subjected to a 1000 ns production run. The generated trajectories were utilized to compute the radius of gyration, end-to-end distance, and radial distribution function (RDF) between terminal groups. These calculations facilitated the elucidation of the local structure of branching PJPs between/among polymer chain ends and between PAP and the terminal groups of PI. To control the system temperature, the Nosé–Hoover thermostat^77,78^ was employed with a coupling constant of 1 ps. The pressure was maintained at 1 bar during both equilibration and production runs using the Berendsen barostat and Parrinello–Rahman barostat.^79^ For equilibration, a time constant of 5 ps and a compressibility of 4.5 × 10^–5^ bar^–1^ (4.5 × 10^–10^ Pa^–1^) were employed. The Verlet cutoff scheme with a cutoff radius of 1.2 nm was utilized to construct the neighbor list in the all-atom MD simulations. Hydrogen bond (HB) lengths were constrained using the LINCS algorithm.^80^ A time step of 2 fs was employed for the all-atom MD simulations. The particle mesh Ewald method^81^ was employed to calculate the electrostatic interactions.

## 3 Results

### 3.1 Equilibration of *cis*-1,4-Polyisoprene Chains: All-Atom MD

In this section, we present the results obtained from our all-atom MD simulations. We focus on investigating various properties of the polymer chains, including end-to-end distances, radii of gyration, mean square internal distances, and rotational time correlation functions. These analyses are conducted for the 13 systems described in Table 1. To assess the equilibrated states of the polymer chains, we specifically analyze the end-to-end distance (*R*_ee_) and radius of gyration (*R*_g_). The time-dependent profiles of *R*_ee_ and *R*_g_ as functions of simulation time are illustrated in Figure S3 (provided in the Supporting Information). Notably, throughout the production run of each system, we observe that *R*_ee_ and *R*_g_ remain stable, indicating that the polymer chains attain a state of equilibrium, with and without dipeptide. Furthermore, we compute the mean square end-to-end distance (⟨*R*_ee_^2^⟩) and mean square radius of gyration (⟨*R*_g_^2^⟩) for the equilibrated systems. The calculated ratio of ⟨*R*_ee_^2^⟩ to ⟨*R*_g_^2^⟩ is provided in Table 2. Notably, the observed ratio, which is close to 6 (Table 2), confirms the characteristic behavior of random Gaussian coil conformations exhibited by the polyisoprene chains. The Kuhn length *b* characterizing a polymer is precisely defined as the quotient of the mean square end-to-end distance ⟨*R*_ee_^2^⟩ and the maximum size when the polymer chain is fully extended *R*_max_.

1 b=⟨Ree2⟩RmaxRmax=bNRB

**Table 2:** Mean-Square End-to End Distance, ⟨*R*_ee_^2^⟩ [nm^2^], and Mean Square Radius of Gyration, ⟨*R*_g_^2^⟩ [nm^2^], Kunh Length, *b* [nm], and Number of Kunh Segments, *N*_RB_, for All Melt Systems at 360 K and 1 bar

system	type of alpha terminal	⟨*R*_ee_^2^⟩		⟨*R*_g_^2^⟩		⟨*R*_ee_^2^⟩/⟨*R*_g_^2^⟩		*b* [nm]		*N*_RB_	
		*P* = 0 wt %	*P* = 1 wt %	*P* = 0 wt %	*P* = 1 wt %	*P* = 0 wt %	*P* = 1 wt %	*P* = 0 wt %	*P* = 1 wt %	*P* = 0 wt %	*P* = 1 wt %
_H_PI_H_		8.73 ± 0.25		1.43 ± 0.02		6.11		0.80		13.50	
_ω_PI_α1_		9.93 ± 0.30	10.03 ± 0.34	1.55 ± 0.02	1.56 ± 0.03	6.39	6.42	0.91	0.92	12.03	11.89
_ω_PI_α3_	ester	9.67 ± 0.28	9.66 ± 0.27	1.54 ± 0.03	1.54 ± 0.03	6.27	6.27	0.85	0.85	13.28	13.33
_ω_PI_α5_		9.36 ± 0.27	9.30 ± 0.28	1.49 ± 0.02	1.48 ± 0.02	6.28	6.27	0.84	0.83	13.30	13.38
_ω_PI_α2_		9.60 ± 0.29	9.73 ± 0.32	1.50 ± 0.02	1.52 ± 0.03	6.38	6.41	0.88	0.89	12.45	12.36
_ω_PI_α4_	hydroxy	9.56 ± 0.28	9.53 ± 0.35	1.52 ± 0.03	1.51 ± 0.03	6.31	6.30	0.86	0.86	12.90	12.94
_ω_PI_α6_		9.00 ± 0.30	9.10 ± 0.28	1.44 ± 0.03	1.45 ± 0.02	6.23	6.26	0.81	0.82	13.59	13.42

The variables *b*, representing the Kuhn length of the polymer chain, *N*_RB_, denoting the number of Kuhn segments, and *R*_max_, indicating the fully extended length of the polymer chain. Our investigation has involved the estimation of both the Kuhn length and the number of Kuhn segments for the polymer chain within each melt system, with detailed results presented in Table 2. The derived values for the Kuhn lengths exhibit close alignment with experimental data.^82^

The chain conformations in each melt system is characterized by mean square internal distances ⟨*R* ^2^(*n*)⟩ (Figure S4 of Supporting Information). The mean square internal distances ⟨*R* ^2^(*n*)⟩ are averaged over all segments of size *n* = *i* – *j* along the chains where *i* < *j* ∈ [1, *N*] are monomer indices. The data confirmed that we have performed sufficiently long simulations for each melt system. Therefore, the chains have moved several times of their own size.

The end-to-end vector autocorrelation function *C*(*t*) was computed for each melt system, serving as a measure of the correlation between the end-to-end vectors over simulation time. The calculation was performed using the following equation

2 $$C\left(t\right)=\left\langle \frac{R\left(t\right)\cdot R\left(0\right)}{R\left(0\right)\cdot R\left(0\right)}\right\rangle$$

where the vectors **R**(0) and **R**(*t*) represent the end-to-end vectors at time *t* = 0 and time *t*, respectively, while the angle bracket denotes an ensemble average. The obtained *C*(*t*) values for the 13 systems, namely, PI_0‑to‑VI_ and PI_I‑to‑VI_ + P, are depicted in Figures 2a and S5. Additionally, we determined the rotational relaxation time by fitting the time correlation function using the simple exponential function

3 $$C\left(t\right)=C\left(0\right)\;\exp\left[-\left(\frac{t}{\tau_{rot}}\right)\right]$$

**Figure 2:**
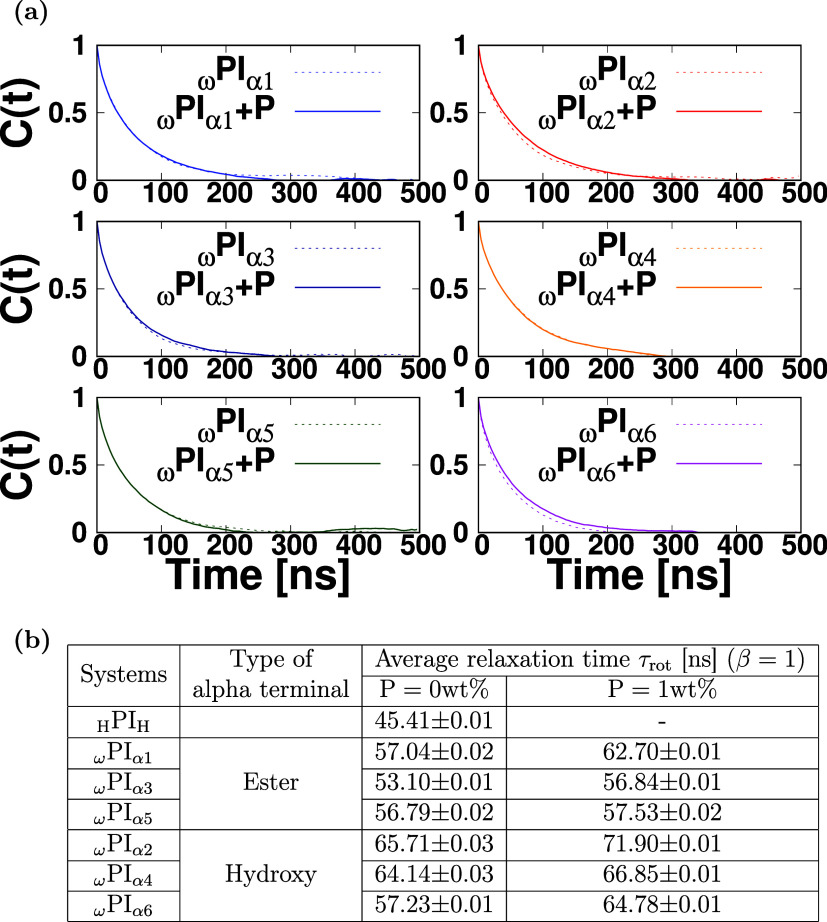
Rotational correlation function vs time for each polyisoprene melt system in the *NVT* ensemble. In (a), the solid and dashed lines correspond to the systems with and without PAP, respectively. (b) Rotational relaxation time τ_rot_. The rotational correlation function *C*(*t*) is fitted using the exponential function to determine the rotational relaxation time τ_rot_.

The rotational correlation function *C*(*t*) is also fitted using the Kohlrausch–Williams–Watts (KWW) stretched exponential function to determine the rotational relaxation time τ_rot_ and the stretching exponent β and the values are shown in Table S1 (Table S1 is given in Supporting Information). From the comparison between Figure 2b (β = 1) and Table S1 (β < 1), we can see that the average relaxation times τ_rot_ obtained by using β = 1 and β < 1 are similar, although the rotational relaxation times estimated by using stretched exponential functions are always respectively smaller than the ones by using simple exponential decay functions.

We have estimated the single-chain dipole moment autocorrelation function ϕ­(*t*) for each melt system by using the following equation

4 ϕ(t)=⟨μ(t)·μ(0)⟩

where the vectors **μ**(0) and **μ**(*t*) represent the dipole moment of a single chain at time *t* = 0 and time *t*, respectively, while the angle bracket denotes an ensemble average. The single-chain total dipole moment autocorrelation function ϕ_total_(*t*) is decomposed into four parts i.e., ϕ_PI,PI_(*t*), ϕ_α*n*,α*n*_(*t*), ϕ_PI,α*n*_(*t*), ϕ_α*n*,PI_(*t*), and these terms are expressed by

5 $$\phi_{PI,PI}\left(t\right)=\left\langle \mu_{PI}\left(t\right)\cdot \mu_{PI}\left(0\right)\right\rangle$$

6 $$\phi_{\alpha n,\alpha n}\left(t\right)=\left\langle \mu_{\alpha n}\left(t\right)\cdot \mu_{\alpha n}\left(0\right)\right\rangle$$

7 $$\phi_{PI,\alpha n}\left(t\right)=\left\langle \mu_{PI}\left(t\right)\cdot \mu_{\alpha n}\left(0\right)\right\rangle$$

8 $$\phi_{\alpha n,PI}\left(t\right)=\left\langle \mu_{\alpha n}\left(t\right)\cdot \mu_{PI}\left(0\right)\right\rangle$$

We have illustrated the single-chain total dipole moment autocorrelation function, denoted as ϕ_total_(*t*), along with its constituent elements in Figures 3, S6, and S7 (Figures S6 and S7 are provided in the Supporting Information). Because the behaviors of ϕ­(*t*) of the six systems (_ω_PI_α*n*_(*n* = 1···6)) are very similar, we only show the ϕ­(*t*)’s of _ω_PI_α1_ and _ω_PI_α2_ in Figure 3, as representative systems with α-terminals containing Ester type (*n*:odd number) and Hydroxy type (*n*:even number).

**Figure 3:**
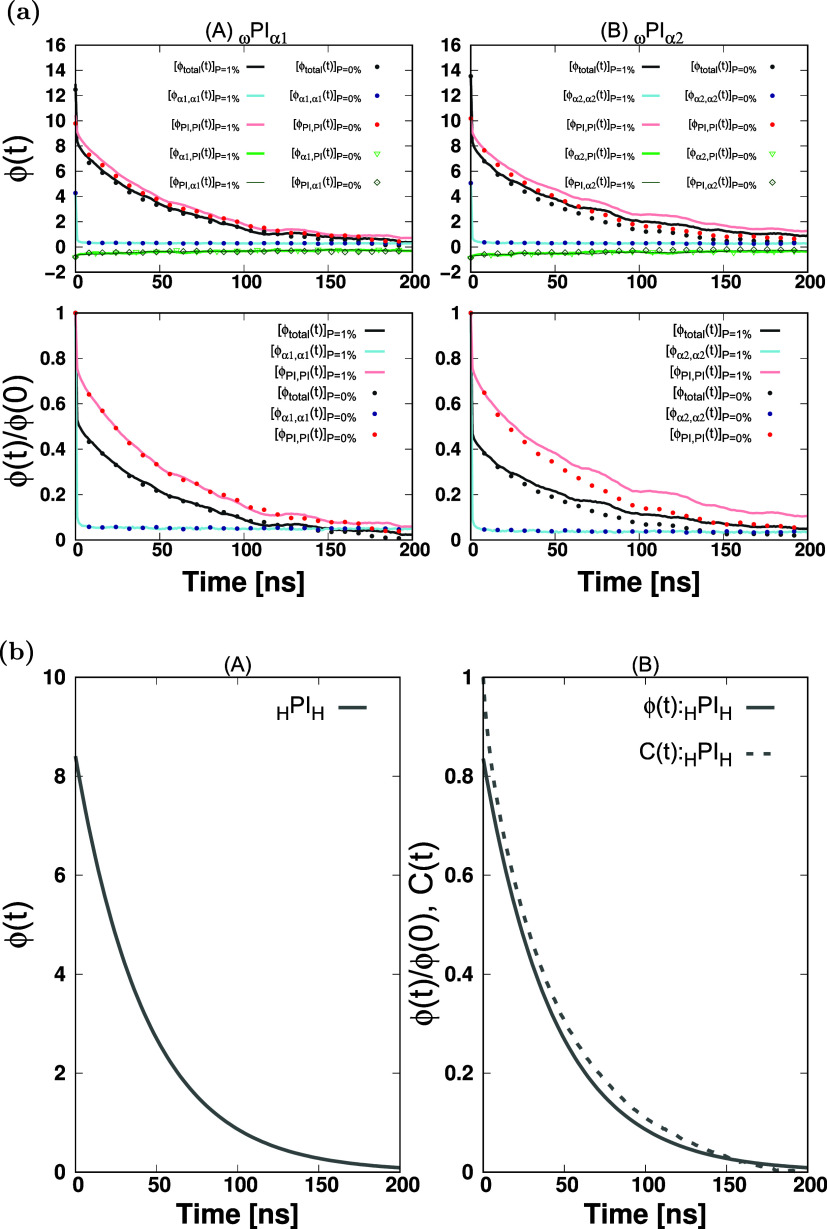
Single-chain dipole moment autocorrelation functions vs time plot for PI_0,I,II_ and PI_I,II_ + P melt systems. The decomposition of the single-chain total dipole moment autocorrelation function, denoted as ϕ_total_(*t*), involves a partition into four distinct components: ϕ_PI,PI_(*t*), ϕ_α*n*,α*n*_(*t*), ϕ_PI,α*n*_(*t*), ϕ_α*n*,PI_(*t*).

In a pure PI system, we can expect that ϕ­(*t*) shows almost the same behavior as that of *C*(*t*) because the direction of the dipole-moment vector in a monomer is aligning along the backbone chain; therefore, the sum of all the dipoles in a chain is proportional to the end-to-end vector of a single chain. As shown in Figure 3, we can confirm that the behavior of ϕ­(*t*) almost follows that of *C*(*t*).

In _ω_PI_α*n*_, on the other hand, as seen from Figures 2 and 3, ϕ­(*t*) does not follow *C*(*t*), especially at around *t* ≃ 0. Namely, ϕ­(*t*) of _ω_PI_α*n*_ exhibits an initial abrupt decay and then exponential decay. The difference in the relaxation behavior between ϕ­(*t*) and *C*(*t*) comes from the polar terminal, i.e., α*n*-terminal. The α1 and α2-terminals have relatively large dipoles (2.25 ± 0.03 D and 2.69 ± 0.03 D, respectively) compared to the magnitude of the total dipole moment vector observed as the sum of the dipole moment vectors in a main chain in equilibrium (3.19 ± 0.05 D and 3.20 ± 0.04 D), and the direction of the dipole moment vector does not necessarily align along the backbone chain direction. As seen from Figure 3, this notable initial abrupt decline of ϕ_total_(*t*) arises predominantly from the abrupt initial decay of ϕ_α*n*,α*n*_(*t*) and the antagonistic effects contributed by ϕ_PI,α*n*_(*t*) and ϕ_α*n*,PI_(*t*). On the other hand, the behavior exhibited by ϕ_PI,PI_(*t*) aligns closely with that of the end-to-end vector autocorrelation function *C*(*t*). These analyses indicate that the role of the polar terminal can make a crucial contribution to the single-chain dipole autocorrelation function ϕ­(*t*), and one should be careful to compare ϕ­(*t*) with the end-to-end vector autocorrelation function *C*(*t*).

### 3.2 Rouse Mode Analysis

We carry out the Rouse mode analysis on the all-atom chains as the coarse-grained chains composed of 12 Rouse beads. According to the Rouse model,^83^ the normal coordinate **X**_*p*_ for free chain ends is defined as

9 $$X_{p}\left(t\right)=\overset{N_{RB}}{\underset{j=1}{\sum }}\sqrt{\frac{2}{N_{RB}}}\;\cos\left(\frac{\left(j-1/2\right)p\pi }{N_{RB}}\right)R_{j}\left(t\right)$$

where *N*_RB_ is the number of Rouse beads in a chain and **R**_*j*_ represents the position of the *j*-th Rouse bead.^84–87^ In the Rouse chain with free ends, the relationship of τ_*p*_/τ_R_ = 1/*p* ^2^ is theoretically expected. Here, τ_R_(≡τ_1_) is the longest chain relaxation time.

The Rouse mode analysis for polymer chain where the chain ends are completely fixed can be performed by using the following normal coordinate^88^

10 $$X_{p}\left(t\right)=\frac{1}{N_{RB}}\int_{0}^{N_{RB}}\sin\left(\frac{\left(p-1/2\right)n\pi }{N_{RB}}\right)R_{n}\left(t\right)\;dn$$

In the fixed chain ends case, the relationship of τ_*p*_/τ_R_ = 1/(*p* – 1/2)^2^ is theoretically expected.

We have computed the time autocorrelation function ⟨**X**_*p*_(*t*)·**X**_*p*_(0)⟩/⟨**X**_*p*_(0)·**X**_*p*_(0)⟩ corresponding to the different Rouse modes, i.e., *p* = 1, 2, 3, 4, 5, and 6, respectively, as obtained from the present all-atom MD simulations. We have shown these time autocorrelation functions in Figure S8 of the Supporting Information. In Table S2 (Table S2 is given in the Supporting Information), we have shown the chain relaxation time τ_1_ for the first Rouse mode (*p* = 1) for each melt system. We have also shown τ_rot._ evaluated by the fitting of a simple exponential decay function (β = 1) to the rotational correlation function *C*(*t*) for each melt system in Figure 2b. We observed a similar trend for the chain relaxation time of first Rouse mode τ_1_ and the rotational relaxation time τ_rot._. This statement can be supported by the ratio τ_R_/τ_rot_(≃1) shown in Table S2.

In accordance with the predictions by the Rouse theory,^83^ the anticipated scaling behavior of relaxation times τ_*p*_ corresponding to each normal mode *p* is outlined. The theoretical framework posits that for unconfined chain ends, the scaling relationship is given by τ_*p*_/τ_R_ = 1/*p* ^2^, while for chain ends held fixed, the relationship transforms to τ_*p*_/τ_R_ = 1/(*p* – 1/2)^2^.^88^ Figure 4 graphically illustrates the dependence of the ratio τ_*p*_/τ_*R*_ on the normal mode *p*. The determination of polymer chain relaxation times τ_*p*_ in each melt system involves fitting the time autocorrelation function ⟨**X**_*p*_(*t*)·**X**_*p*_(0)⟩/⟨**X**_*p*_(0)·**X**_*p*_(0)⟩ into a single exponential function exp­(−*t*/τ_*p*_). If Rouse scaling holds true, all curves depicted in Figure 4 are expected to align on a common trajectory corresponding to τ_*p*_/τ_R_ = 1/*p* ^2^ for free chain ends and τ_*p*_/τ_R_ = 1/(*p* – 1/2)^2^ for fixed chain ends. In the instance of PI_0_ (_H_PI_H_), where polyisoprene chains possess free chain ends, adherence to the Rouse scaling is evident (as represented by the gray color symbol “+” in Figure 4 for Rouse modes *p* = 1, 2, 3, 4, 5, and 6). Similarly, for the PI_I‑to‑VI_ and PI_I‑to‑VI_ + P melt systems, the polyisoprene chains also conform to the free-end Rouse scaling, which also means that any distortion in higher modes is not induced by such intermittent associations among chain ends. In Figure 4b, we have shown the chain relaxation time τ_R_ for each melt system. These results of Figure 4a,b indicate that the systems (PI_I‑to‑VI_ and PI_I‑to‑VI_ + P) having end-group associations still obey the scaling of the Rouse model with free ends but only the scale of the relaxation time is prolonged by the intermittently restricted dynamics of chain-ends. In addition, from the table in Figure 4b we can see that the prolonging of the relaxation time can be enhanced by PAP. In summary, judging from the data within the present analyses, the intermittent association among α-terminals does not change the Rouse dynamics behavior but does change the time scale of Rouse relaxation, and PAP can enhance the prolong of the relaxation.

**Figure 4:**
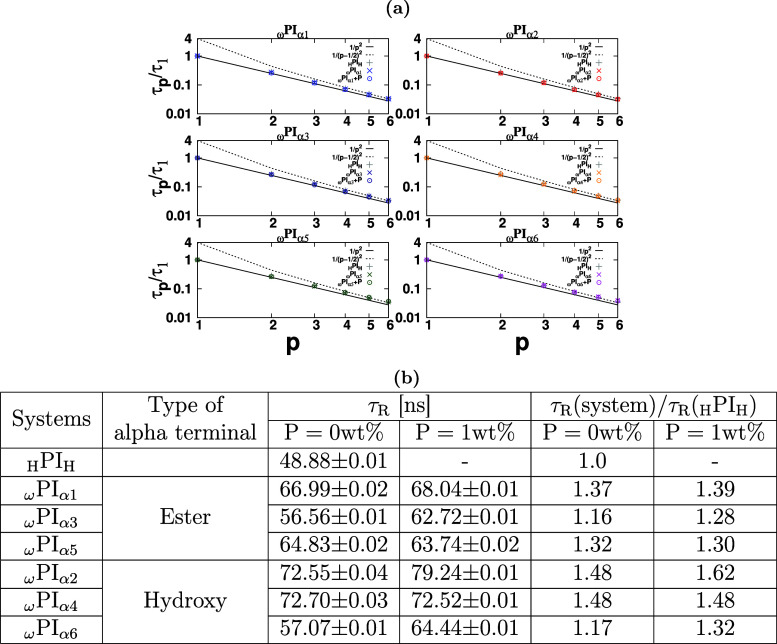
In (a), the figure encapsulates the relaxation times associated with distinct normal modes *p* of the chains, offering insights into the dynamic behavior of the melt system. Complementarily, (b) focuses on the chain relaxation time τ_*p*_ for the first Rouse mode (*p* = 1), providing a comprehensive perspective on the temporal evolution. The time autocorrelation function ⟨**X**_*p*_(*t*)·**X**_*p*_(0)⟩/⟨**X**_*p*_(0)·**X**_*p*_(0)⟩ undergoes fitting with a simple exponential function, serving as a crucial tool in extracting the chain relaxation time τ_*p*_.

### 3.3 Association of PAP with Ester- and Hydroxy-Terminated Polyisoprene Chains

The present section is dedicated to exploring the associations between various terminal groups, including the ω-terminal and ω-terminal, PAP and ω-terminal, and PAP and α-terminal, in both PI_0‑to‑VI_ and PI_I‑to‑VI_ + P melt systems. We present snapshots of the PI_0‑to‑VI_ + P melt systems obtained after a 1 μs production run in Figure 5.

**Figure 5:**
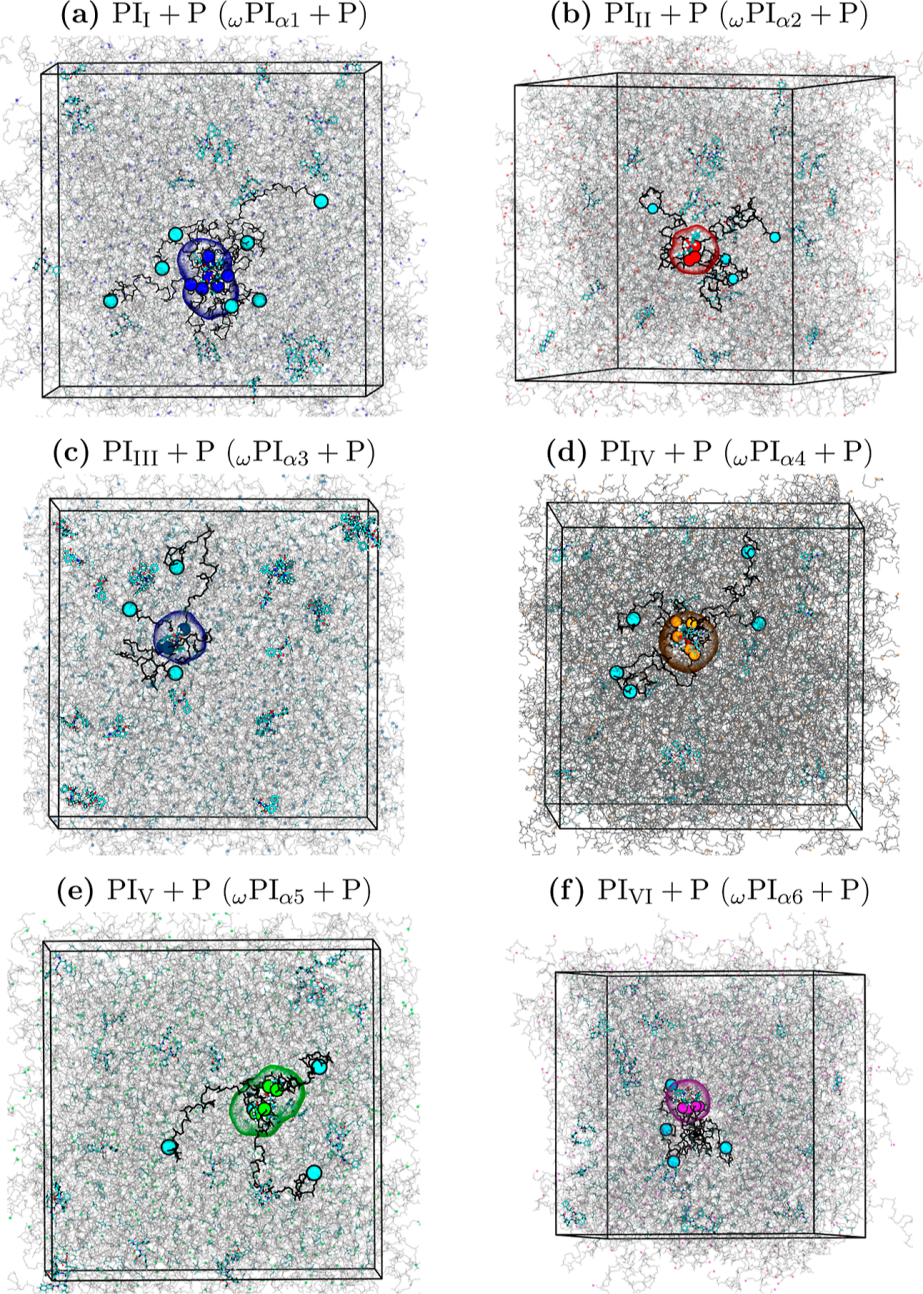
Final configurations of the following PI melt systems obtained after 1 μs production-run of all-atom MD simulations: (a) PI_I_ + P, (b) PI_II_ + P, (c) PI_III_ + P, (d) PI_IV_ + P, (e) PI_V_ + P, and (f) PI_VI_ + P. In the snapshots, the DMA group of ω terminals (DMA group and two *trans*-1,4-isoprene) is visualized in cyan color, while α1, α2, α3, α4, α5, and α6 terminals are indicated by blue, red, web-blue, orange, green, and magenta colors, respectively. The backbone carbon and hydrogen atoms are illustrated in gray color. Additionally, the PJPs of α1 around the peptide, α2 around the peptide, α3 around the peptide, α4 around the peptide, α5 around the peptide, and α6 around the peptide are identified using blue, red, web-blue, orange, green, and magenta colors, respectively.

In Figure 5, we have highlighted the PJPs of α1 around PAP, α2 around PAP, α3 around PAP, α4 around PAP, α5 around PAP, and α6 around PAP using blue, red, web-blue, orange, green, and magenta colors, respectively. The observations clearly demonstrate that the α terminals of hydroxy and ester-terminated polyisoprene chains are associated with the backbone atoms of PAP.

In this study, we conducted an investigation into the association of α and ω terminal groups with PAP and also explored the impact of PAP on α-terminal and α-terminal interactions. To achieve this, we utilized RDFs to analyze the spatial relationships. Specifically, we computed the RDFs between the centers of mass of terminal groups belonging to different polyisoprene chains. Additionally, we calculated the RDFs for the distribution of α and ω terminals around PAP. The RDFs *g*_αβ_(*r*) are defined by the following equation

11 $$g_{\alpha \beta }\left(r\right)=\frac{{\left\langle \rho_{\beta }\left(r\right)\right\rangle }_{local,\alpha }}{{\left\langle \rho_{\beta }\left(r_{c}\right)\right\rangle }_{\alpha }}$$

where the quantity ${\left\langle \rho_{\beta }\left(r\right)\right\rangle }_{local,\alpha }$ denotes the average density of type β particles in the vicinity of type α particles at a distance *r*. It is defined as

12 $${\left\langle \rho_{\beta }\left(r\right)\right\rangle }_{local,\alpha }=\frac{1}{N_{\alpha }\left(\delta_{\alpha \beta }+1\right)}\overset{N_{\alpha }}{\underset{i_{\alpha }=1}{\sum }}{\left\langle \rho_{\beta }\left(r\right)\right\rangle }_{local,i_{\alpha }}$$

In eq 12, ${\left\langle \rho_{\beta }\left(r\right)\right\rangle }_{local,i_{\alpha }}$ denotes the local density of type β particles in the vicinity of the *i*-th type α particle, computed at a distance *r*. The definition is given by

$$\left\langle \rho_{\beta }\left(r\right)\right\rangle {\left\langle \rho_{\beta }\left(r\right)\right\rangle {\left\langle \rho_{\beta }\left(r\right)\right\rangle }_{local,i_{\alpha }}=\sum_{j_{\beta }=1}^{N_{\beta }}{\;}^{\prime }\delta (r}_{i_{\alpha }j_{\beta }}-r)$$ 13

In eq 13, the prime notation in the sum indicates that the summation is performed over distinct particles when α and β are different, i.e., ∑′ is $\overset{N_{\beta }}{\underset{j_{\beta }=1}{\sum }}$ . However, when α and β represent the same particle type, the summation excludes the contribution from the *i*_α_-th particle itself, denoted as $\overset{N_{\beta }}{\underset{j_{\beta =1},j_{\beta }\neq i_{\alpha }}{\sum }}$ . This distinction ensures that self-interactions are not counted when calculating the local density. Regarding eq 11, the denominator on the right-hand side, ${\left\langle \rho_{\beta }\left(r_{c}\right)\right\rangle }_{\alpha }$ , represents the average density of particle type β within a sphere with radius *r*_c_ centered around the α particle. It is mathematically defined as follows

14 $${\left\langle \rho_{\beta }\left(r_{c}\right)\right\rangle }_{\alpha }=\frac{1}{N_{\alpha }}\overset{N_{\alpha }}{\underset{i_{\alpha }=1}{\sum }}{\left\langle \rho_{\beta }\left(r_{c}\right)\right\rangle }_{i_{\alpha }}$$

The density of particle type β within a sphere with radius *r*_c_ centered around the *i*-th type α particle, denoted by ⟨ρ_β_(*r*_c_)⟩_*i*α_, is mathematically defined as follows in eq 14

15 $${\left\langle \rho_{\beta }\left(r_{c}\right)\right\rangle }_{i_{\alpha }}=\frac{1}{V}\int_{0}^{r_{c}}{\left\langle \rho_{\beta }\left(r\right)\right\rangle }_{local,i_{\alpha }}4\pi r^{2}\;dr$$

In eq 15, *V* represents the volume of the sphere centered around the *i*-th type α particle and the radius *r*_c_ of the sphere is selected to be half of the simulation box length. The variables *N*_α_ and *N*_β_ represent the total number of α and β particles, respectively. The Kronecker delta function δ_αβ_ is defined as δ_αβ_ = 1 when α = β and δ_αβ_ = 0 when α ≠ β. The inclusion of the factor (δ_αβ_ + 1) in the denominator is introduced to avoid double counting when α = β. This ensures an accurate computation of the density of particle type β within the specified sphere around the *i*-th type α particle.

In this study, we have conducted a comprehensive analysis of the RDFs involving the center of mass of the DMA group and the phenyl ring of PAP as well as the RDFs among DMA groups of ω terminals, denoted as [Phenyl]-[DMA], [DMA]-[DMA] in _ω_PI_α1‑to‑α6_ (here and hereafter, [X] stands for a chemical functional group X, and Y around X is symbolically expressed by [X]–[Y]). These RDFs are shown in Figure 6. Notably, in the case of PI_0‑to‑VI_ + P, the local density of phenyl around DMA is significantly lower compared to DMA around DMA. As a result, the association between PAP and DMA is weaker than that observed between DMA groups.

**Figure 6:**
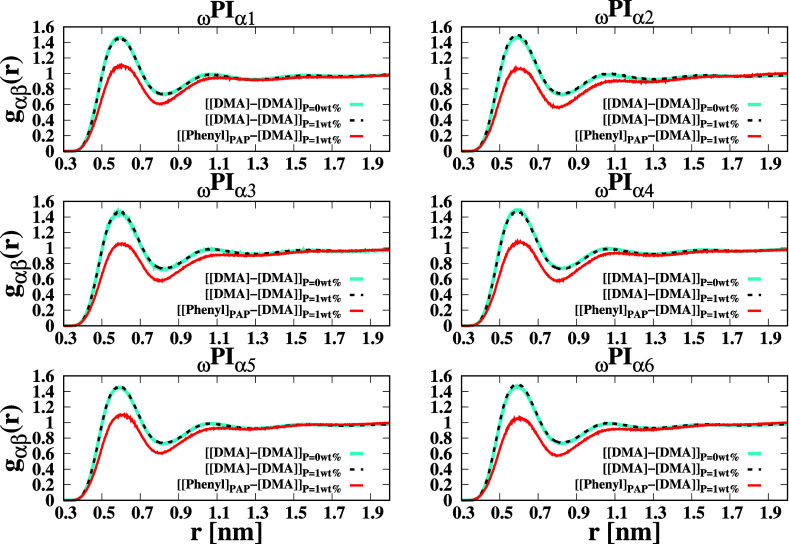
RDFs of DMA around DMA and DMA around dipeptide molecules in different cis polyisoprene systems. The center of mass of the phenyl residue of the PAP molecule is denoted by “[Phenyl]”, and the center of mass of the DMA residue of the ω terminal is denoted by “[DMA]”. The RDFs are calculated for various combinations of α and β particles represented by [Phenyl] and [DMA]. The RDFs of β particles around α particles are symbolically expressed as [α] – [β]. The RDF curves are plotted using the following colors: solid red for ([Phenyl_PAP_] – [DMA]) with *P* = 1 wt %, solid cyan for [DMA] – [DMA] with *P* = 1 wt %, and dashed black for [DMA] – [DMA] with *P* = 0 wt %.

In our investigation, we further explored the RDFs of specific molecular interactions within the polyisoprene melt systems. Specifically, we estimated the RDFs of [[C]_C=O_] around [[C]_C=O_] (representing the RDF of carbon atom of carbonyl groups around carbon atom of carbonyl groups of ester-terminated polyisoprene chains, denoted as α_2*m*–1_), and [[O]_C=O_] around [[NH]_PAP_] (representing the RDF of the oxygen atom of the carbonyl group of ester terminal groups around the hydrogen atom bonded to the nitrogen atom of the backbone of PAP) in PI_I,III,V_ + P. Furthermore, we examined the RDFs of [[O]_OH_] – [[H]_OH_] (representing the hydrogen atom of hydroxy groups of hydroxy-terminated polymer chains around the oxygen atom in another OH group) and [[NH]_PAP_] – [O_OH_] (representing the oxygen atom of hydroxy groups of hydroxy-terminated polymer chains around NH of PAP, i.e., α_2*m*_ around hydrogen atom bonded to nitrogen atom of backbone of PAP) for PI_II,IV,VI_ + P. Figure 7 presents the RDFs of interest for the PI_I,III,V_ + P systems. Notably, we observed a distinctive peak at a distance of 0.2 nm in the [[NH]_PAP_] – [[O]_C=O_] RDF, indicating a strong association between the carbonyl groups of ester-terminated polyisoprene chains and the backbone of PAP. This association is facilitated by hydrogen bonding, providing compelling evidence for the establishment of PJPs between PAP and the ester terminals of polyisoprene chains. The local density of [[O]_C=O_] surrounding [[NH]_PAP_] exhibits a significantly higher value when compared to the local densities of [[C]_C=O_] surrounding [[C]_C=O_]. The position of first peak of [[NH]_PAP_] – [[O]_OH_] RDF in _ω_PI_α2,α4,α6_ is observed at 0.2 nm, providing evidence of HB formation between [[NH]_PAP_] and [α2], [[NH]_PAP_] and [α4], and [[NH]_PAP_] and [α6], which, in turn, facilitates the creation of PJPs between hydroxy-terminated polyisoprene chains and PAP. The intensity of the first RDF peak of [[NH]_PAP_] – [[O]_OH_] is remarkably higher than that of [[O]_OH_] – [[H]_OH_], providing strong evidence for the stronger association of [PAP] and [α] in comparison to [α_2*m*_] and [α_2*m*_]. We investigated the coordination patterns of [[O]_OH_] around [[NH]_PAP_] and [[O]_OH_] around [[O]_OH_] in different polyisoprene systems. We found that there are three coordination shells of [[O]_OH_] around [[NH]_PAP_] but only two coordination shells of [[O]_OH_] around [[O]_OH_]. This indicates that the PJP between [PAP] and hydroxy terminal groups is more ordered compared to the PJPs between [[O]_OH_] and [[O]_OH_]. Furthermore, we analyzed the intensity of the first RDF peak of [[NH]_PAP_] – [[O]_OH_] and [[NH]_PAP_] – [[O]_C=O_] and observed that the association between [PAP] and hydroxy-terminated polyisoprene chains is significantly stronger than that in ester-terminated polyisoprene chains. Additionally, the second RDF peak intensity of [[NH]_PAP_] – [[O]_OH_] is more pronounced than that of [[NH]_PAP_] – [[O]_C=O_], indicating that the PJPs between hydroxy terminals and [PAP] are more structured than those between ester terminals and [PAP]. In the case of PI_II,IV,VI_ + P systems, the presence of three narrower and one broader coordination shells of [[O]_OH_] around [[NH]_PAP_] confirms the formation of highly networked PJPs between [PAP] and hydroxy-terminated PI chains. Conversely, for PI_I,III,V_ + P systems, one narrower and two broader coordination shells of [[O]_C=O_] around [[NH]_PAP_] suggest the formation of less networked structures between [PAP] and ester-terminated PI chains. Furthermore, we observed that the local density of [[O]_C=O_] around [[NH]_PAP_] in the first coordination shell is significantly larger than that of [[O]_C=O_] around [[O]_C=O_], implying that the PJP between [PAP] and ester terminal groups is very stable and highly ordered, whereas the PJP between ester terminal groups is less stable.

**Figure 7:**
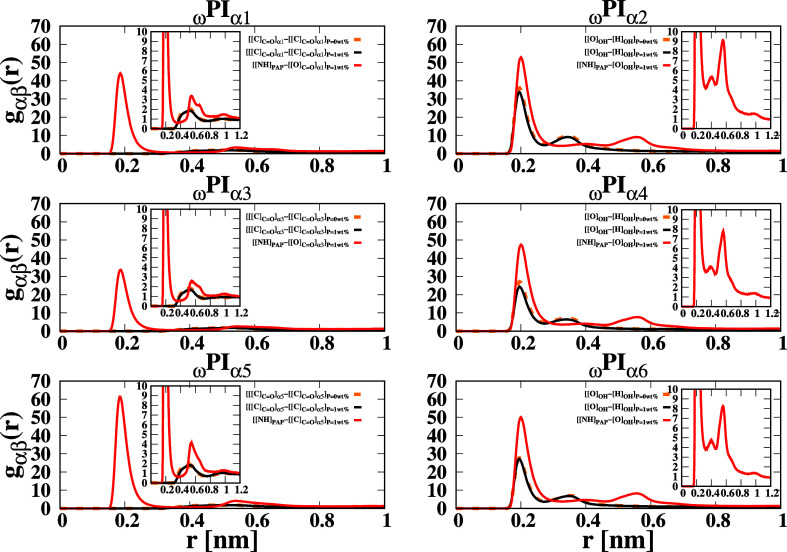
RDFs of terminals around terminals and terminals around dipeptide molecules in 12 types of cis polyisoprene systems. “[[O]_C=O_]” represents the oxygen atom in the ester group in α1, α3, and α5 terminals, and [[O]_OH_] and [[H]_OH_] stand for the oxygen and hydrogen atoms of the hydroxy group in α2, α4, and α6 terminals. The RDFs are calculated by considering [[O]_C=O_], [Phenyl], [[NH]_PAP_], [DMA], [[O]_OH_], [[H]_OH_] as α and β particles in the expression *g*_αβ_(*r*). The RDFs represent the interactions between β particles and α particles and are symbolically expressed as [α] – [β]. The RDF curves are drawn using different colors: solid black for ([[C]_C=O_] – [[C]_C=O_]), solid red for ([[NH]_PAP_] – [[O]_C=O_]), solid black for ([[O]_OH_] – [[H]_OH_]), and dashed orange for ([[C]_C=O_] – [[C]_C=O_]) and ([[O]_OH_] – [[H]_OH_]). Solid lines represent systems with *P* = 1 wt %, while dashed lines correspond to systems with *P* = 0 wt %.

Figure 8 presents the RDFs of terminal groups in the presence and absence of PAP. A notable observation is that the association between [α] and [α] is significantly diminished in the presence of a dipeptide. The first peak of the [α] – [α] RDFs becomes broader when PAP is present. Consequently, PAP molecules show a preference for solvation by α terminals, which promotes the formation of multiple PJPs. These results shed light on the impact of PAP on the interterminal interactions within the system and highlight the preferential solvation behavior induced by the presence of PAP.

**Figure 8:**
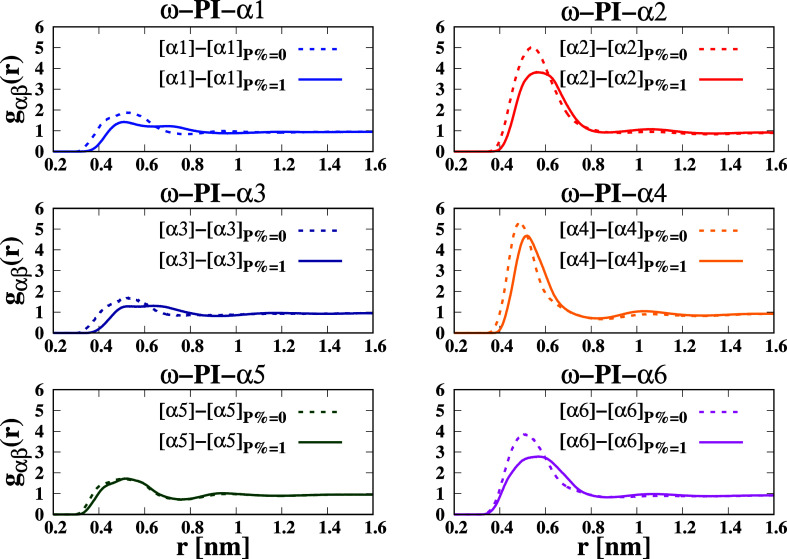
RDFs of the terminals around terminals in the 12 types of cis polyisoprene systems. “[α]” stands for the center of mass of α terminal of *cis*-1,4-polyisoprene chain. As α and β in *g*_αβ_, [α*m*] is considered. The RDF of β around α (symbolically expressed by [α] – [β]) are drawn by the following colors: solid blue for ([α1] – [α1]), solid red for [α2] – [α2], solid dark-blue for [α3] – [α3], orange color for [α4] – [α4], dark-green color for [α5] – [α5], and magenta color for [α6] – [α6]. Solid lines correspond to *P* = 1 wt % and dashed lines are for *P* = 0 wt %.

We have investigated the solvation structure encompassing the backbone of the PAP. To achieve this, we employ eq 16 to compute the running coordination numbers (RCNs) delineating the spatial arrangement of surrounding species around the peptide backbone. The coordination number is defined as

16 $$n_{\alpha \beta }=4\pi \rho_{\beta }\int_{r_{1}}^{r_{2}}r^{2}g_{\alpha \beta }\left(r\right)\;dr$$

In this context, *n*_αβ_ denotes the number of type β atoms surrounding species α, confined within a radial shell extending from *r*_1_ to *r*_2_. Here, ρ_β_ represents the number density of β within the system, while *g*_αβ_(*r*) stands for the RDF. The latter provides the ratio of the local density of β around α to the bulk density of β. For the specific calculation of the first solvation shell coordination number, *r*_1_ is set to zero, signifying the immediate vicinity of the species α, and *r*_2_ corresponds to the position of the first minimum observed in the RDF. This method captures the nuanced spatial arrangement of atoms surrounding a central species. The RCNs, *n*_αβ_, elucidating the interatomic associations of terminals around terminals and terminals around dipeptide molecules within 12 distinct cis polyisoprene systems, are graphically depicted in Figure 9 for comprehensive visualization and analysis.

**Figure 9:**
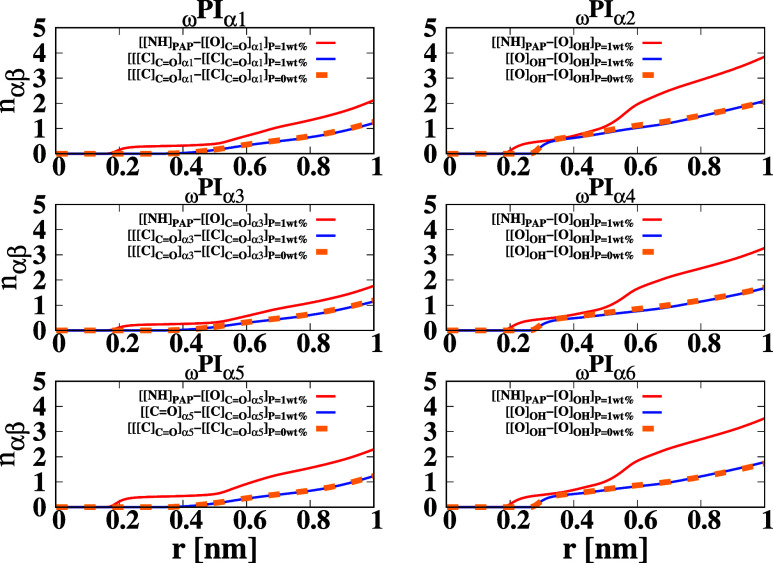
RCNs, *n*_αβ_(*r*), of terminals around terminals and terminals around dipeptide molecules in 12 types of cis polyisoprene systems. “ $\left[O\right]\left[O\right]{\left[O\right]}_{C=O}]$ ” represents the oxygen atom in the ester group in α1, α3, and α5 terminals, and [[O]_OH_] stands for the oxygen atom of the hydroxy group in α2, α4, and α6 terminals. The RCNs are calculated by considering [[O]_C=O_], [[NH]_PAP_], [[O]_OH_] as α and β particles in the expression *g*_αβ_(*r*). The RCN curves are drawn using different colors: solid black for ([[C]_C=O_] – [[C]_C=O_]), solid red for ([[NH]_PAP_] – [[O]_C=O_]), solid blue for ([[O]_OH_] – [[O]_OH_]), and dashed orange for ([[C]_C=O_] – [[C]_C=O_]) and ([[O]_OH_] – [[O]_OH_]). Solid lines represent systems with *P* = 1 wt %, while dashed lines correspond to systems with *P* = 0 wt %.

From the analysis of Figure 9, it is evident that the population of α terminals within the first, second, and third coordination shells surrounding dipeptide molecules (red line) is markedly greater compared to the corresponding population around individual α terminals. This difference arises from the distinctive structural features of the dipeptide molecule, which encompasses polar N-terminal, C-terminal, and peptide bond functionalities (−NH–C­(=O)−). Consequently, the affinity of α terminals for interaction with PAP is substantially heightened in the context of the dipeptide, resulting in a notably higher number of α terminals bound to the PAP in comparison to the population around individual α terminals. Therefore, the numerical density of α terminal groups in the vicinity of the dipeptide is significantly elevated relative to that around isolated α terminals. In the present investigation, we utilized the single linkage algorithm^89–91^ to analyze the formation of clusters. Specifically, we focused on characterizing the “encountering-state” of an end-group of a polymer chain with other end-groups belonging to different polymer chains or PAP molecules. This state occurs when the distance between two end-groups from different chains or between the dipeptide molecule and the end-group falls below a threshold distance *r*_th_ at a given time step. By identifying these encounters, we establish a relationship between the end-groups involved in the association. Additionally, if one of the two ends is in an encountering state with a different end-group, the third end-group is also considered to be in an encountering state with the first two end-groups or the dipeptide and end-group. This process is repeated for the newly added end-group, and when no further end-groups can be added, we determine the size *s* of the encountering end-groups by counting the total number of participating end-groups. By evaluating the number (*n*_*s*_) of encountering end-groups of size *s*, we gain insights into the encountering behavior of the end-groups and their interactions with the dipeptide molecules within the system.

The notation used to distinguish between the two cases of encountering end-groups is as follows: when the end-groups in an encountering state only contain a single type of end-group, α type, we use the notation *n*_*s*_^αα^. On the other hand, when the encountering end-groups involve two different types of ends, α and β, the notation *n*_*s*_^αβ^ is applied. To evaluate the number of encountering end-groups of a particular size *s*, we perform this analysis for *K* times during the last 100 ns of the 1000 ns simulation trajectories. Here, *K* corresponds to the number of frames present in the 100 ns simulation trajectories, i.e., *n*_*s*_^(αβ)^(*k*) where *k* = 1, ..., *K* = 50,000. By utilizing the obtained values *n*_*s*_^(αβ)^(*k*), we define the encountering-event-fraction *f*_enc_^(αβ)^(*s*) for a specific size *s* as follows

17 $$f_{enc}^{\left(\alpha \beta \right)}\left(s\right)=\frac{1}{M}\frac{1}{K}\overset{K}{\underset{k=1}{\sum }}n_{s}^{\left(\alpha \beta \right)}\left(k\right)$$

where the parameter *M* represents the total number of chains in the system. Figure 10 illustrates the encountering-event-fraction *f*_enc_^(αβ)^(*s*) as a function of the encountering-event size *s* for each melt system. Specifically, *f*_enc_^(αβ)^(*s* = 2) denotes the probability of an end group encountering another end group, indicating that any end group of any chain is linked with an end group of another chain, resulting in the existence of 100% end groups as dimers. As the size of the encountering-event increases, the encountering-event-fraction gradually decreases. In the absence of a dipeptide (i.e., *P* = 0 wt %) in ester-terminated polyisoprene melt systems (_ω_PI_α1,α3,α5_), we predominantly observe encountering-end-groups of size two, with a few of size three, and only very few of size four. However, in the presence of a dipeptide (i.e., *P* = 1 wt %), larger-sized encountering-end-groups also form in _ω_PI_α1,α3,α5_ + P. Moreover, peptide molecules promote encountering of peptide with end groups of sizes two, three, and four in _ω_PI_α1,α3,α5_ + P. In the case of *P* = 0 wt % systems, namely, _ω_PI_α2_, _ω_PI_α4_ and _ω_PI_α6_ systems, we observe not only encountering-events of size two and three but also larger-sized encountering-events due to the strong HB interactions between [α2] and [α2], [α4] and [α4], and [α6] and [α6] terminals. Furthermore, for *P* = 1 wt % systems, i.e., _ω_PI_α2,α4,α6_ + P, the fraction of encountering-events significantly increases, and we observe encountering-events of size larger than six. Notably, the encountering-events between dipeptide and hydroxy terminals are more pronounced compared to those involving ester terminals. While we can observe encountering events between end-groups and dipeptide-end groups with certain fractions, the stability of the formed clusters, which we refer to as stable physical junctions, cannot be determined.

**Figure 10:**
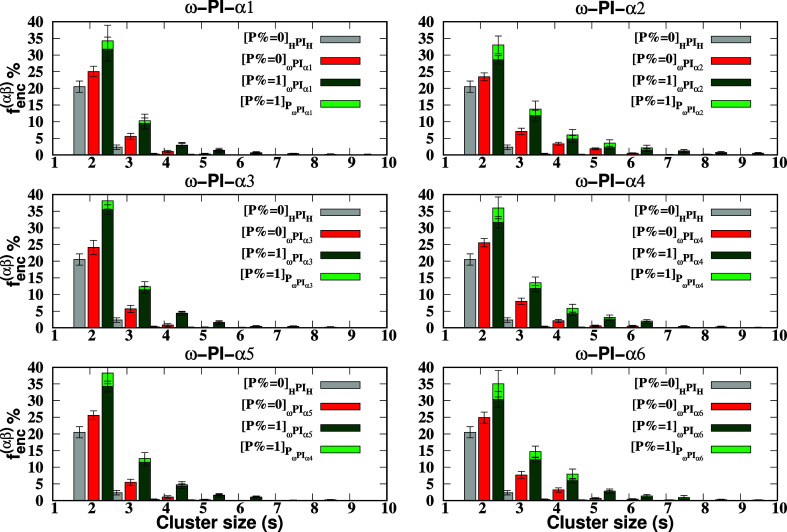
Fraction of end-group encounters with a size *s* for PI_0‑to‑VI_ and PI_I‑to‑VI_ + P systems, where the cut threshold length *r*_th_ is set to 0.6 nm. It is important to note that $\overset{\infty }{\underset{s=1}{\sum }}f_{enc}^{\left(\alpha \beta \right)}\left(s\right)=1$ for each system, ensuring that the total fraction of encounters accounts for all sizes, and the fractions for *s* = 1 are not shown in the figure.

The potentials of mean force (PMFs) are widely utilized to investigate the stability of clusters, as demonstrated in various studies.^60,61,92–106^ Accordingly, in this study, we have computed the PMFs between the terminal groups and also between the dipeptide and the terminal groups, employing the following equation

18 $$W\left(r\right)=-k_{B}T\;\log\left(g\left(r\right)\right)$$

where *k*_B_ represents the Boltzmann constant in kJ mol^–1^/K, *T* is the temperature of the system, and *g*(*r*) denotes the RDF between the terminal groups. Figure 11 illustrates the PMFs between the two terminal groups as a function of their distance. Notably, for each scenario, the PMF profile exhibits a distinct minimum, which we have denoted as the contact minimum (CM). Specifically, in the case of PI_0_, we have observed a shallow minimum for contact pairs involving the two terminals [ISO] (ISO represents the H-terminated *cis*-1,4-isoprene residue) and [ISO] at a distance of 0.6 nm.

**Figure 11:**
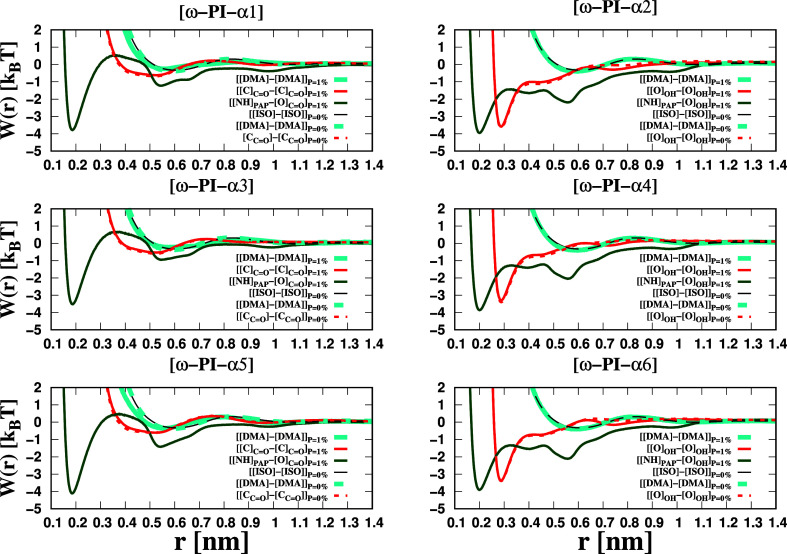
PMFs, *W*(*r*), calculated using the equation *W*(*r*) = −*k*_B_ *T* log­(*g*(*r*)). Here, *k*_B_ represents the Boltzmann constant with units of kJ mol^–1^/K, and *T* is the temperature of the system. The PMFs were determined based on the RDF *g*(*r*) between the terminal groups. The magnitude of error bars in each PMF profile is less than 0.5*k*_B_ *T*.

In the _ω_PI_α1_, _ω_PI_α3_, and _ω_PI_α5_ melt systems, we observed shallow minima for contact pairs between [[C]_C=O_] and [[C]_C=O_] at a distance of 0.5 nm and between [DMA]- and [DMA]-end groups at a distance of 0.6 nm. However, the interaction free energy of the contact pairs between [ISO]- and [ISO]-terminals in the _H_PI_H_ system (shown by the black line in Figure 11), as well as between [[C]_C=O_] and [[C]_C=O_] in the _ω_PI_α1,α3,α5_ systems, was found to be within the order of thermal energy. Consequently, these contact pairs cannot be considered as stable configurations. In contrast, the _ω_PI_α2_, _ω_PI_α4_ and _ω_PI_α6_ systems exhibit distinct sharp minima for contact pairs between [[O]_OH_] and [[O]_OH_], indicating significantly higher stabilities of these associations against thermal energy. Consequently, the stabilities of the associations between [α2] and [α2], [α4] and [α4], and [α6] and [α6] are considerably greater than those of [ISO] and [ISO], [DMA] and [DMA], [α1] and [α1], [α3] and [α3], and [α5] and [α5] terminals’ associations. These observations are consistent with the findings reported in our previous work.^22,62^ In the presence of a dipeptide, we observed highly stable contact pairs and less stable solvent-shared pairs and solvent-separated pairs between [[NH]_PAP_] and [[O]_C=O_] in the _ω_PI_α1,α3,α5_ + P melt systems at distances of 0.2, 0.55, and 1.00 nm, respectively. Similarly, in the _ω_PI_α2,α4,α6_ + P systems, we observed stable contact pairs and less stable solvent-assisted pairs, solvent-shared pairs, and solvent-separated pairs between [[NH]_PAP_] and [[O]_OH_] at distances of 0.2, 0.4, 0.55, and 1.00 nm, respectively. To assess the formation of stable clusters, we considered the value of |*W*| at the CM, which should be significantly greater than the thermal energy. Hence, we set the criteria for cluster formation as |*W*| > 2*k*_B_ *T* at the CM. The dissociation barrier height for the CM of end groups corresponds to the free energy difference between the CM and the first maximum of the PMF.^107^ Notably, the dissociation barriers for [[NH]_PAP_] and [[O]_C=O_] contact pairs are considerably higher than those for [[NH]_PAP_] and [[O]_OH_] contact pairs. From the analysis of Figure 11, the values of dissociation barriers between [O] and [O] CM are 7.6 kJ/mol (2.5*k*_B_ *T*), 7.9 kJ/mol (2.6*k*_B_ *T*), and 7.8 kJ/mol (2.6*k*_B_ *T*) for _ω_PI_α2_, _ω_PI_α4_, and _ω_PI_α6_, respectively. In the case of *P* = 1 wt %, the values of dissociation barriers for [[NH]_PAP_] and [[O]_C=O_] CM are 12.94 kJ/mol (4.3*k*_B_ *T*), 12.5 kJ/mol (4.2*k*_B_ *T*), and 13.73 kJ/mol (4.6*k*_B_ *T*) for _ω_PI_α1_ + P, _ω_PI_α3_ + P, and _ω_PI_α5_ + P melt systems. As for [NH]_PAP_] and [[O]_OH_] contact pairs, the estimated values of dissociation barriers are 7.523 kJ/mol (2.5*k*_B_ *T*), 7.73 kJ/mol (2.6*k*_B_ *T*), and 7.706 kJ/mol (2.6*k*_B_ *T*) for _ω_PI_α2_ + P, _ω_PI_α4_ + P, and _ω_PI_α6_ + P melt systems.

Utilizing the PMF results, we establish a criterion for determining stable associations between two terminal groups. A stable association is deemed to occur when the PMF (*W*) between two terminal groups satisfies the inequality: (|*W*(*r*_CM_)| > 2*k*_B_ *T*), with *r*_CM_ representing the distance at the CM. Such stable associations are capable of forming a “cluster”. To assess the number of terminal groups participating in a cluster, we apply two criteria: (i) the distance criterion mentioned in eq 17 and (ii) the criterion for stable association (|*W*(*r*_CM_)| > 2*k*_B_ *T*). The resulting number of clusters with a size *s* is denoted as *n*_*s*_^(αβ)^(*k*;|*W*(*r*_CM_)| > 2*k*_B_ *T*), where α and β refer to terminal groups such as [[O]_C=O_] or [[C]_C=O_] or [OH] or the backbone of [PAP]. The meaning of the superscripts α and β on *n*_*s*_ aligns with their usage in the context of the number of encountering end groups. For example, *n*_*s*_^(PAP,OH)^ represents the number of clusters with a size *s* containing a single [PAP] molecule and [OH] residues of the terminals, whereas *n*_*s*_^(OH,OH)^ indicates the number of clusters with a size *s* composed solely of OH terminal groups. By employing these numbers, we define the cluster-formation-fraction *f*_cluster_^(αβ)^(*s*) as follows

19 $$f_{cluster}^{\left(\alpha \beta \right)}\left(s\right)=\frac{1}{M}\frac{1}{K}\overset{K}{\underset{k=1}{\sum }}n_{s}^{\left(\alpha \beta \right)}\left(k;|W\left(r_{CM}\right)|>2k_{B}T\right)$$

In Figure 12, the cluster-formation-fraction *f*_cluster_^(αβ)^(*s*) is depicted. In the absence of PAP (*P* = 0 wt %), the systems _H_PI_H_, _ω_PI_α1_, _ω_PI_α3_, and _ω_PI_α5_ show a complete absence of cluster formation by the terminal groups, while the encountering-events between the end-groups persist, as clearly shown in Figure 10. However, in the presence of PAP, clusters between [PAP] and ester terminals are formed with size *s* = 2. On the other hand, in the case of *P* = 0 wt %, for _ω_PI_α2_, _ω_PI_α4_, and _ω_PI_α6_, clusters with sizes two to six are observed, and no clusters of size *s* ≥ 7 exist. However, in the presence of PAP, the fraction of cluster formation becomes more pronounced, and clusters of larger sizes are also present. For _ω_PI_α2_ + P, clusters between peptide and hydroxy terminal groups are observed, ranging in size from two to nine, while no clusters of size *s* ≥ 8 exist in _ω_PI_α4_ + P and _ω_PI_α6_ + P. In the case of _ω_PI_α4_ + P and _ω_PI_α6_ + P, clusters of size two to five are perceived. It is worth noting that a similar trend was previously reported in our research.^61^

**Figure 12:**
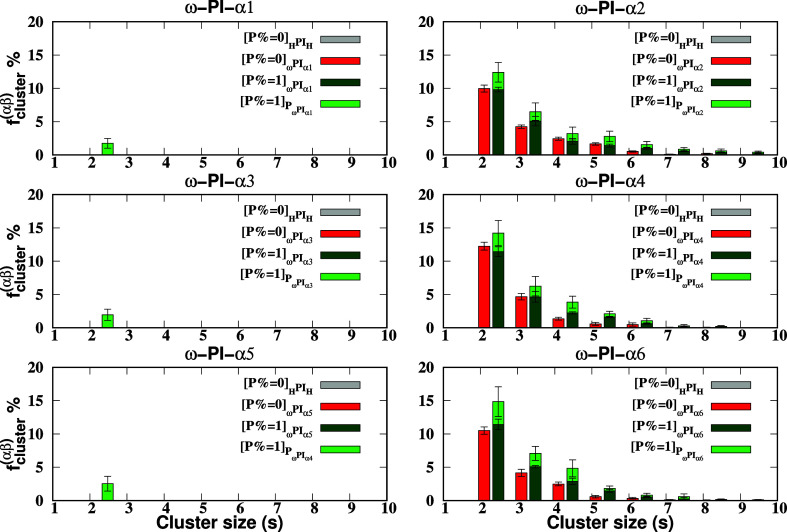
Cluster-formation-fraction as a function of cluster size *s* is depicted for the PI_0‑to‑VI_ and PI_I‑to‑VI_ + P systems. The plot showcases the probability of stable clusters composed of terminal groups of varying sizes. In addition to the criterion employed in Figure 10, an additional condition is applied, requiring the PMF between the terminal groups to exceed a threshold value compared to the thermal energy. This additional criterion is utilized to assess whether the terminal groups can indeed form stable clusters, importantly, the sum of all cluster-formation-fractions $\overset{\infty }{\underset{s=1}{\sum }}f_{cluster}^{\left(\alpha \beta \right)}\left(s\right)=1$ for each system, ensuring comprehensive coverage of all terminal groups. The fractions for *s* = 1 are not displayed in the figure.

### 3.4 Survival Probability of Terminal Groups around the Backbone of the Dipeptide

The survival probability *P*(τ) represents the probability of observing a group of particles remaining within a specific spherical region, characterized by a radius (*r*_th_^sp^), and centered at the geometrical center of the selected group.^108,109^ To obtain the survival probability, we have employed the following equation.

20 $$P\left(\tau \right)=\frac{1}{M_{tot}}\overset{M_{tot}}{\underset{t=1}{\sum }}\frac{N\left(t,t+\tau \right)}{N\left(t\right)}$$

Where *N*(*t*) represents the count of terminals present within a specific spherical region that has a radius of *r*_th_^sp^ and is centered at the geometrical center of the selected group. This count is determined at a particular time instant *t*, and the terminals must be assigned to a cluster to be considered in this analysis. Second, *N*(*t*, *t* + τ) corresponds to the number of terminals that persistently remain within the same spherical region during the time interval from *t* to *t* + τ. The parameter τ signifies the time duration between the configurations that we analyze. Lastly, to accurately evaluate *P*(τ), we consider a total of *M*_tot_ time steps during our study. These variables collectively enable us to assess the survival probability and understand the dynamics of the terminals within the specified spherical region over time. To establish the appropriate parameter *r*_th_^sp^ governing the region of interest for assessing *P*(τ) with regard to [ISO] around [ISO], [[C]_C=O_] around [[C]_C=O_], [[O]_OH_] around [[O]_OH_], [[O]_OH_] around [[NH]_PAP_], and [[O]_C=O_] around [[NH]_PAP_], we have used the RDFs [ISO] – [ISO], [[C]_C=O_] – [[C]_C=O_], and [[O]_OH_] – [[O]_OH_] (“[[O]_OH_]” represents the oxygen atom of the hydroxy group of hydroxy terminated polyisoprene chains), [[NH]_PAP_] – [[O]_OH_] and [[NH]_PAP_] – [[O]_C=O_] (Figure 7). From the results of RDFs, we have chosen as the radius of selected regions that of the first coordination shells of [ISO] around [ISO], [C]_C=O_ around [C]_C=O_ and [O]_OH_ around [O]_OH_ and [O]_C=O_ around [NH]_PAP_ and [O]_OH_ around [NH]_PAP_. We estimated the survival probability using MDAnalysis.^110^ In Figure 13, we present the survival probability of terminal groups around terminal groups in the polyisoprene systems with PAP, with _H_PI_H_ serving as a reference. The observed order of decay times is as follows: _H_PI_H_ < _ω_PI_α3_ + P ≈ _ω_PI_α1_ + P < _ω_PI_α5_ + P ≪ _ω_PI_α4_ + P < _ω_PI_α6_ + P < _ω_PI_α2_ + P. Notably, the survival probabilities exhibited slower decay in hydroxy-terminated polyisoprene chains: _ω_PI_α2_ + P, _ω_PI_α4_ + P and _ω_PI_α6_ + P, compared to ester-terminated polyisoprene chains, namely, _ω_PI_α1_ + P, _ω_PI_α3_ + P and _ω_PI_α5_ + P. By fitting these survival probability curves *P*(τ) with the KWW stretched exponential function, we estimated the characteristic decay times (τ_sp_) as follows: 6.05 ps (pure PI), 17.43 ps (_ω_PI_α1_ + P), 73.07 ps (_ω_PI_α2_ + P), 14.92 ps (_ω_PI_α3_ + P), 46.97 ps (_ω_PI_α4_ + P), 23.08 ps (_ω_PI_α5_ + P), and 57.64 ps (_ω_PI_α6_ + P). These results indicate a considerably slower exchange rate of terminal groups within the first coordination shell in hydroxylated polyisoprene chains compared to pure PI and ester-terminated polyisoprene chains. The relaxation time of hydroxy terminals around hydroxy terminals is longer than that of ester terminals around ester terminals. Furthermore, we determined the order of decay times (τ_sp_) of *P*(τ) of α around PAP as follows: [[NH]_PAP_] and [α4] < [[NH]_PAP_] and [α6] < [[NH]_PAP_] and [α2] < [[NH]_PAP_] and [α1] < [[NH]_PAP_] and [α3] < [[NH]_PAP_] and [α5]. Consequently, the decay times of terminal groups around PAP are significantly slower compared to terminal groups around terminal groups, indicating that the PJPs between PAP and alpha terminal groups are more structured and stable than those of α terminal groups. Moreover, the decay times of ester terminal groups around PAP are substantially larger compared to hydroxy terminal groups around PAP, suggesting that PJPs between PAP and ester terminal groups are more stable compared to PJPs between PAP and hydroxy terminal groups. Further details, including decay times and stretching exponents with error bars, are provided in Table 3.

**Figure 13:**
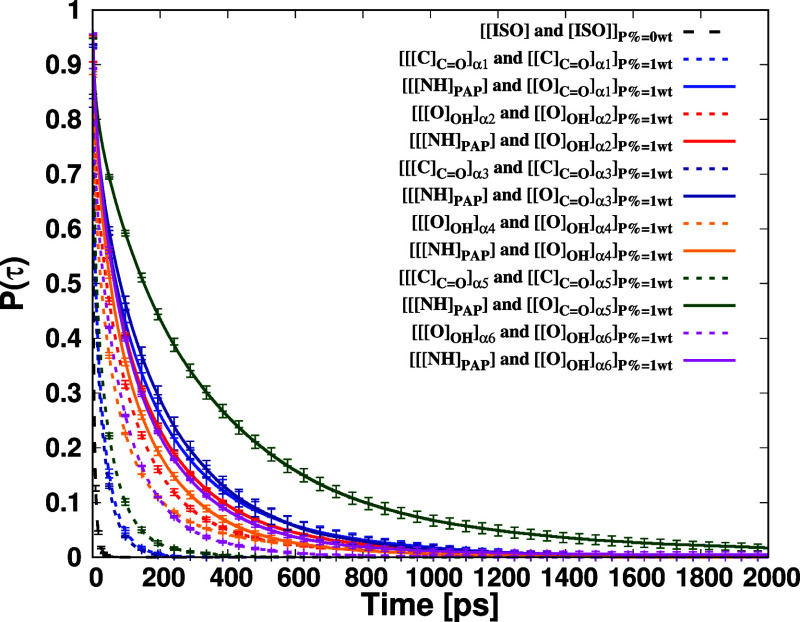
Survival probabilities *P*(τ) pertaining to terminal groups residing within the first coordination shell of the terminal group. The survival probability calculations were derived from the final 10 ns of 1000 ns independent MD simulations. The determination of error bars was accomplished using the block-average method. Notably, each survival probability was computed by averaging across the available time windows within the specified interval, ranging from 1 to 1000 ps.

**Table 3:** Results Obtained by Fitting the Survival Probability *P*(τ) Data Using the KWW Stretched Exponential Function^a^

systems	type of alpha terminal	average survival time (τ_sp_)/[ps]			stretching exponents (β_sp_)		
		*P* = 0 wt %	*P* = 1 wt %		*P* = 0 wt %	*P* = 1 wt %	
		[α] and [α]	[α] and [α]	[[NH]_PAP_] and [α]	[α] and [α]	[α] and [α]	[[NH]_PAP_] and [α]
_H_PI_H_		6.05 ± 0.05			0.66		
_ω_PI_α1_		17.71 ± 0.05	17.43 ± 0.04	146.14 ± 0.23	0.60	0.62	0.71
_ω_PI_α3_	ester	16.37 ± 0.05	14.92 ± 0.04	170.51 ± 0.30	0.60	0.61	0.78
_ω_PI_α5_		24.48 ± 0.08	23.08 ± 0.06	296.00 ± 0.54	0.59	0.58	0.76
_ω_PI_α2_		64.96 ± 0.30	73.07 ± 0.23	117.48 ± 0.13	0.68	0.62	0.68
_ω_PI_α4_	hydroxy	46.45 ± 0.18	46.97 ± 0.10	97.56 ± 0.10	0.67	0.54	0.69
_ω_PI_α6_		56.22 ± 0.25	57.64 ± 0.22	112.70 ± 0.23	0.66	0.64	0.69

a This fitting procedure allows us to calculate the average survival time τ_sp_ and β_sp_ parameters, providing valuable insights into the relaxation dynamics of the system under investigation.

### 3.5 Self-Diffusion Coefficient of the Polyisoprene Chain

This section focuses on the exploration of the diffusion behavior exhibited by polymer melts. To assess the mean square displacement (MSD), the final 100 ns of the 1000 ns trajectories were considered. Figure 14 graphically presents the time-evolution of the MSD of the center of mass for individual chains. The self-diffusion coefficients, *D* of polyisoprene chains within each melt system were quantified employing the Einstein relation, defined as follows.

21 $$D=\lim_{t\to \infty }\;\frac{\left\langle {\left(R_{cm}\left(t\right)-R_{cm}\left(0\right)\right)}^{2}\right\rangle }{6t}$$

**Figure 14:**
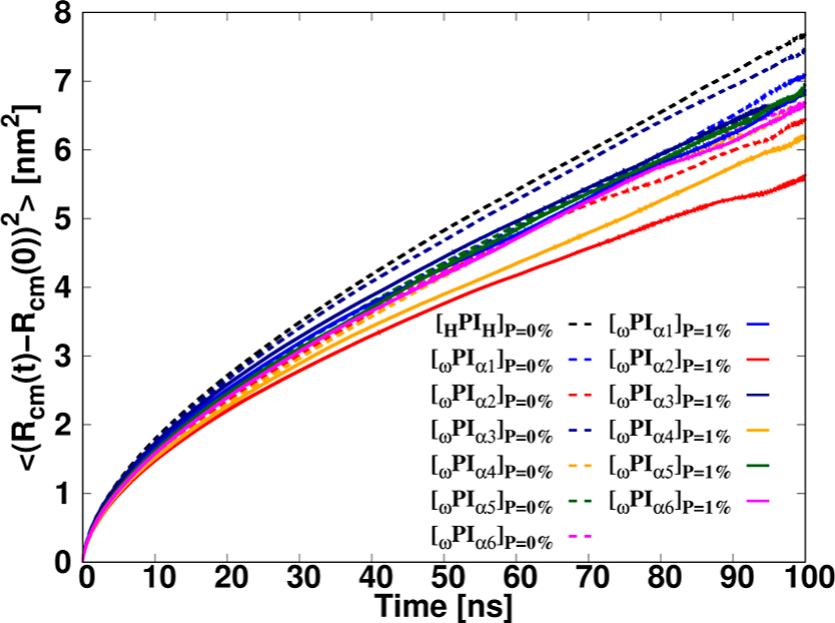
Temporal variation of MSD for the centers of mass of *cis*-1,4-polyisoprene chains in each melt system.

The MSD of the center of mass of a polymer chain, denoted as ⟨(**R**_cm_(*t*) −**R**_cm_(0))^2^⟩, was calculated, and the resulting self-diffusion coefficients are presented in Table 4. In the presence of PAP, a substantial reduction in the motility of polyisoprene chains was observed. Consequently, the single-chain mobilities in pure-PI and _ω_PI_α1,α3,α5_ + P systems exhibited higher motilities compared to those of _ω_PI_α2,α4,α6_ + P melt systems. Notably, the introduction of P = 1 wt % resulted in the formation of HBs between [PAP] and [α_*n*_] (*n* = 1, 2, ···, 6) terminals, leading to a substantial decline in the mobility of polyisoprene chains. Interestingly, the _ω_PI_α2_ molecular chains in the _ω_PI_α2_ + P system exhibited the lowest motility among all the systems studied. The lowest mobility in _ω_PI_α2_ + P is attributed to the formations of stable clusters of α2 with PAP, which is consistent with the notable elevation in peak intensities observed within the first, second, and third coordination shells of [[NH]_PAP_]–[[O]_OH_], in contrast to the corresponding intensities identified in the _ω_PI_α4_ + P and _ω_PI_α6_ + P configurations (Figure 7).

**Table 4:** Self-Diffusion Coefficients *D* of a Polyisoprene Chain

systems	type of alpha terminal	self-diffusion coefficients *D*/[10^–^^6^ cm^2^/s]	
		*P* = 0 wt %	*P* = 1 wt %
_H_PI_H_		0.1072 ± 0.064	
_ω_PI_α1_		0.0960 ± 0.021	0.0910 ± 0.023
_ω_PI_α3_	ester	0.1030 ± 0.026	0.0950 ± 0.029
_ω_PI_α5_		0.1000 ± 0.036	0.0990 ± 0.019
_ω_PI_α2_		0.0900 ± 0.03	0.0767 ± 0.028
_ω_PI_α4_	hydroxy	0.0940 ± 0.023	0.0830 ± 0.021
_ω_PI_α6_		0.0934 ± 0.063	0.0927 ± 0.023

### 3.6 Dynamic Behavior of HBs Involving α Terminal Groups around the Dipeptide

Within this section, our focus lies in the exploration of HB dynamics between PAP and hydroxy α terminals as well as ester α terminals within the polymer melt systems. To achieve this, we calculate the widely recognized HB time correlation function *C*_HB_(*t*) for pairs *i*, *j* of hydrogen-bonded polymer chains. This correlation function’s definition is based on the previous seminal works.^111–114^

22 $$C_{HB}^{x}\left(t\right)=\left\langle \frac{\sum h_{ij}\left(t_{0}\right)h_{ij}\left(t_{0}+t\right)}{\sum {\left(h_{ij}\left(t_{0}\right)\right)}^{2}}\right\rangle$$

where *h*_*ij*_(*t*) serves as an indicator of whether a pair *i*, *j* satisfies the geometric criteria for hydrogen bonding at time *t*. Specifically, *h*_*ij*_(*t*) = 1 indicates the presence of a HB, while *h*_*ij*_(*t*) = 0 signifies its absence. The summation is carried out over all possible hydrogen-bonded pairs *i*, *j* of polymer chains, and angular brackets represent an average over different starting times *t*_0_ in the trajectory. In this investigation, we explore the dynamics of HBs formed between PAP and hydroxy α terminals, as well as ester α terminals, in the polymer melt systems. To assess HB dynamics, we calculate the HB time correlation function *C*_HB_(*t*) for pairs *i*, *j* of polymer chains. The superscript x distinguishes two different definitions for measuring *h*_*ij*_(*t*) at future points in time: continuous *C*_HB_^c^(*t*) and intermittent *C*_HB_^I^(*t*). These correlation functions offer distinct insights into HB dynamics. Continuous HB correlation *C*_HB_^c^(*t*) reflects the average time a pair remains intact as a HB before breaking, yielding the continuous lifetime τ_HB_^c^. In contrast, intermittent HB correlation *C*_HB_^I^(*t*) investigates the persistence probability of a HB created at *t* = 0, despite multiple breakings and reformations during the time period [0, *t*]. The corresponding lifetime is known as the intermittent lifetime or HB relaxation time τ_HB_^I^. Using the MDAnalysis package,^110,115^ we estimated the continuous *C*_HB_^c^(*t*) and intermittent *C*_HB_^I^(*t*) HB correlation functions for specific pairs, such as ([[NH]_PAP_], [[O]_C=O_]_α2*m*−1_), ([[NH]_PAP_], [[O]_OH_]_α2*m*_) (*m* = 1, 2, 3), as displayed in Figure 15. Moreover, we determined the HB relaxation time τ_HB_ by fitting the HB auto correlation functions *C*_HB_^c^(*t*) and *C*_HB_^I^(*t*) into the KWW stretched exponential function. The values of τ_HB_ and the stretching exponent β_HB_ are presented in Table 5. The intermittent HB correlation function was fitted into two types of KWW stretched exponential functions, namely $C_{HB}^{\prime I}\left(t\right)=C_{HB}^{\prime I}\left(0\right)\exp\left[-{\left(t/\tau_{HB}^{\prime I}\right)}^{\beta_{HB}^{\prime I}}\right]$ and $C_{HB}^{I}\left(t\right)=P_{HB}^{I}+C_{HB}^{I}\left(0\right)\exp\left[-{\left(t/\tau_{HB}^{I}\right)}^{\beta_{HB}^{I}}\right]$ . The fitted functions are shown in Figure S11, and χ^2^ values are provided in Table S3. Based on the χ^2^ values, *C*_HB_^I^(*t*) yields a better fitting compared to *C*_HB_^′I^(*t*). The estimated values of τ_HB_^I^, stretching exponent β_HB_^I^, and *P*_HB_^I^ are presented in Table 5.

**Figure 15:**
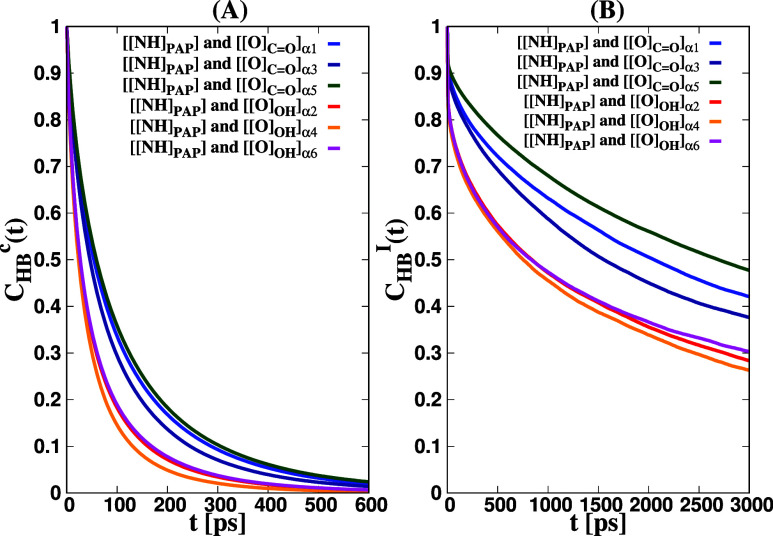
HB correlation functions, specifically the continuous *C*_HB_^c^(*t*) (A) and intermittent *C*_HB_^I^(*t*) (B), for various pairs in the polymer melt systems are illustrated. The pairs investigated include ([[NH]_PAP_], [O]­[O]­[O]_C=O_]_α2*m*−1_), ([[NH]_PAP_], [O]­[O]­[O]_OH_]_α2*m*_) (*m* = 1, 2, 3). Each HB correlation function is represented by a distinctive color, where blue, red, dark-blue, orange, dark-green, and magenta correspond to the pairs mentioned above, respectively.

**Table 5:** HB Relaxation Time τ_HB_ and Stretching Exponent β_HB_ for _ω_PI_α*n*_ Melt Systems (*n* = 1, 2, 3, 4, 5, 6)

systems	type of alpha terminal	continuous		intermittent		
		τ_HB_^c^	β_HB_^c^	τ_HB_^I^	β_HB_^I^	*P*_HB_^I^
_ω_PI_α1_ + P		47.81 ± 0.08	0.687 ± 0.0007	5421.72 ± 13.01	0.4731 ± 0.0015	0.0730 ± 0.0008
_ω_PI_α3_ + P	ester	45.88 ± 0.10	0.679 ± 0.0009	4230.33 ± 9.70	0.512 ± 0.002	0.0789 ± 0.0008
_ω_PI_α5_ + P		54.04 ± 0.078	0.710 ± 0.0007	6453.36 ± 14.1	0.522 ± 0.002	0.0703 ± 0.0005
_ω_PI_α2_ + P		25.13 ± 0.09	0.613 ± 0.001	3646.43 ± 19.83	0.398 ± 0.001	0.1139 ± 0.1139
_ω_PI_α4_ + P	hydroxy	21.08 ± 0.05	0.671 ± 0.001	3344.33 ± 21.66	0.399 ± 0.001	0.1279 ± 0.0015
_ω_PI_α6_ + P		23.57 ± 0.067	0.621 ± 0.001	3191.71 ± 22.81	0.380 ± 0.001	0.0963 ± 0.0014

The relaxation times of HBs follow the order ([[NH]_PAP_], [[O]_OH_]_α6_) < ([[NH]_PAP_], [[O]_OH_]_α4_) < ([[NH]_PAP_], [[O]_OH_]_α2_) < ([[NH]_PAP_], [[O]_C=O_]_α3_) < ([[NH]_PAP_], [[O]_C=O_]_α1_) < ([[NH]_PAP_], [[O]_C=O_]_α5_). These results indicate that the continuous and intermittent HB lifetimes (as shown in Figure 12 and Table 5) are significantly larger for ([[NH]_PAP_], [[O]_C=O_]_α1_), ([[NH]_PAP_], [[O]_C=O_]_α3_), and ([[NH]_PAP_], [[O]_C=O_]_α5_) compared to ([[NH]_PAP_], [[O]_OH_]_α2_), ([[NH]_PAP_], [[O]_OH_]_α4_), and ([[NH]_PAP_], [[O]_OH_]_α6_), mainly because the HB dissociation barriers for ([[NH]_PAP_], [[O]_C=O_]_α2*m*−1_) are significantly larger than those of ([[NH]_PAP_], [[O]_OH_]_α2*m*_) (as observed in Figure 8). Consequently, ester terminal groups play a vital role in promoting the formation of more stable PJPs around PAP molecules. These findings offer valuable insights into the preferential formation of stable HBs within the polymer network and the consequent impact on the overall system’s stability.

## 4 Discussion and Conclusions

In this investigation, we have undertaken a comprehensive study to elucidate the impact of PAP on the equilibrium properties of PI melt systems. These PI systems encompass diverse combinations of dimethyl allyl-(*trans*-1,4-isoprene) (ω) and α terminal groups. To gain in-depth insights, we employed rigorous all-atom MD simulations. Our analyses involved the calculation of crucial equilibrium parameters, including the end-to-end distance *R*_ee_, radius of gyration *R*_g_, and mean square internal distances. Additionally, we explored the spatial arrangements of terminal groups around each other and around PAP using RDFs. Moreover, we investigated the dynamics of the PI chains by analyzing the end-to-end vector autocorrelation function *C*(*t*) and the rotational relaxation time τ_rot_. Furthermore, we examined the self-diffusion coefficients to gain a comprehensive understanding of the chain dynamics in the presence of PAP. Our investigation involved a thorough analysis of Figure 2a, which revealed a distinct slowdown in the rotational relaxation behavior of hydroxy and ester-terminated polyisoprene chains in the presence of PAP molecules. This observation provides strong evidence for the existence of clustering between PAP and the terminal groups of polyisoprene chains. To gain deeper insights, we conducted estimations of the RDFs pertaining to the terminal groups around the terminal group and those surrounding PAP, specifically focusing on [Phenyl]-[DMA], [DMA]-[DMA], [O]_OH_ – [H]_OH_, [[NH]_PAP_] – [[O]_OH_], [[C]_C=O_]–[[C]_C=O_], [[NH]_PAP_]–[[O]_C=O_], [α1] – [α1], [α2] – [α2], [α3] – [α3], [α4] – [α4], [α5] – [α5], and [α6] – [α6] interactions (Figures 6–8). Regarding the *P* = 0 wt % and *P* = 1 wt % cases, we have observed a significantly higher intensity of the first peak in the [[O]_OH_] – [[H]_OH_] RDFs for _ω_PI_α2_, _ω_PI_α4_, and _ω_PI_α6_ compared to [[C]_C=O_] – [[C]_C=O_] in _ω_PI_α1_, _ω_PI_α3_, _ω_PI_α5_. This compellingly indicates a much stronger association between hydroxy terminal groups in comparison to ester terminal groups, as corroborated by the slower decay of survival probability for α2 around α2, α4 around α4, and α6 around α6 terminals (Figure 13). Moreover, the higher intensity of the first peak in the [[O]_OH_] – [[O]_OH_] RDFs for _ω_PI_α2_, _ω_PI_α4_, and _ω_PI_α6_ (Figure S10) exhibits a similar trend, which is in line with previous studies.^22,61,116^

Interestingly, we observed a significant reduction in the local density of [α] around [α] clustering in the presence of PAP (Figure 8), suggesting preferential solvation of PAP by α terminal groups over ω terminal groups. For PAP = 1 wt %, the low intensity and broad first RDF peaks of [Phenyl]–[DMA] in comparison to [DMA]–[DMA] suggest that PAP molecules are weakly associated with ω terminal groups in each melt system. In the following paragraph, we will delve into the molecular details of these aggregates, referred to as PJPs.

Furthermore, the analysis of the first RDF peak of [[NH]_PAP_] – [[O]_C=O_] revealed hydrogen bonding between the carbonyl group of ester terminals and the backbone of PAP (Figure 7), confirming the formation of PJPs between PAP and ester terminal groups of polyisoprene chains. Similarly, the considerable increase in the local density of the first RDF peak of [[NH]_PAP_] – [[O]_OH_] compared to [[O]_OH_] – [[H]_OH_] indicates that PAP molecules facilitate the formation of PJPs with hydroxy terminals of polyisoprene chains. Consequently, PAP molecules are strongly associated with both the α terminal groups of hydroxy and ester-terminated polyisoprene chains. Moreover, the presence of three narrower and one broader coordination shells of [[O]_OH_] around [[NH]_PAP_] confirms the formation of highly networked PJPs between PAP and hydroxy-terminated PI chains, while the presence of one narrow and two broader coordination shells of [[O]_C=O_] around [[NH]_PAP_] indicates the formation of a globular structure between PAP and ester-terminated PI chains. In the _ω_PI_α2_ + P melt system, an enhanced interaction between [[NH]_PAP_] and [[O]_OH_] entities becomes evident, in contrast to the scenarios found in _ω_PI_α4_ + P and _ω_PI_α6_ + P. This phenomenon finds its origin in the distinctive structural compositions of these species. Specifically, the hydroxy-terminated isopentene (α2) incorporates a methylene group as its side chain, while hydroxy-terminated isobutane (α4) and hydroxy-terminated 1,4-*cis*-isoprene (α6) feature a methyl group. Notably, the methyl–methyl interaction entails a heightened steric repulsion compared to the methylene–methylene interaction. Additionally, the linear configuration of hydroxy-terminated isopentene contrasts with the spherical topology exhibited by hydroxy-terminated isobutane (α4) and hydroxy-terminated 1,4-*cis*-isoprene (α6). A similar observation is reported for the interaction between O and H in our previous work.^62^ This phenomenon aligns with a similar claim reported in prior experimental research,^5^ where the spatial organization of proteins in NR was investigated using STORM, revealing their interactions with the α terminal groups of polyisoprene chains, ultimately leading to the formation of aggregates that facilitate the creation of a natural network structure.

We have conducted a comprehensive analysis of the encountering-event-*f*_enc_^(αβ)^(*s*) fraction for terminal groups in 13 different types of melt systems (Figure 10), along with the potentials of the mean force between these terminal groups (Figure 11) and the cluster-formation-fraction of terminal groups (Figure 12). Notably, the encountering-event-fraction of size two is significantly larger than that of other sizes in each melt system. Furthermore, the hydroxy terminal groups of polyisoprene chains promote the formation of larger size encountering-events in the _ω_PI_α2_ + P, _ω_PI_α4_ + P, and _ω_PI_α6_ + P melt systems compared to those observed in the pure PI, _ω_PI_α1_ + P, _ω_PI_α3_ + P, and _ω_PI_α5_ + P systems. The relative order of encountering-event-fractions for dimer, trimer, tetramer, pentamer, and hexamer is *f*_enc(dimer)_^(αβ)^ > *f*_enc(trimer)_^(αβ)^ > *f*_enc(tetramer)_^(αβ)^ > *f*_enc(pentamer)_^(αβ)^ > *f*_enc(hexamer)_^(αβ)^. Remarkably, a similar trend has been observed in our previous work^22,61^ and in hydroxylated polybutadiene.^116^ We have assessed the interaction free energy among the encountered end-groups using the PMFs *W*(*r*). The _H_PI_H_ system exhibits a shallow CM at approximately 0.6 nm in the potential profile. Similarly, in _ω_PI_α1_ + P, _ω_PI_α3_ + P, and _ω_PI_α5_ + P systems, a shallow CM is observed around 0.5 nm, with free energy depths at these CMs comparable to the thermal energy (2.99 kJ/mol at 360 K). Conversely, the _ω_PI_α2_ + P, _ω_PI_α4_ + P, and _ω_PI_α6_ + P melt systems present a sharp CM at approximately 0.3 nm, with free energy depths at these CMs significantly larger than the thermal energy. These findings provide evidence that the association between hydroxy-terminated PI is more stable than that of ester-terminated PI, [ISO]–[ISO], and [ω] – [ω], as corroborated by the survival probabilities for terminal groups. Notably, we observed the presence of CM, solvent shared minimum (SShM), and solvent separated minimum (SSM) between [[NH]_PAP_] and [[O]_OH_] in _ω_PI_α1_ + P, _ω_PI_α3_ + P, and _ω_PI_α5_ + P systems. Conversely, in _ω_PI_α2_ + P, _ω_PI_α4_ + P, and _ω_PI_α6_ + P systems, there exist CM, solvent-assisted minimum (SAM), SShM, and SSM between [[NH]_PAP_] and [[O]_OH_]. This suggests that the hydroxy terminal groups exhibit more ordered arrangements around the backbone of PAP compared to ester terminal groups. Additionally, the dissociation barrier for the CM between [[NH]_PAP_] and [[O]_C=O_] is notably larger than that between [[NH]_PAP_] and [[O]_OH_]. Therefore, the CM between the backbone of PAP and ester terminal groups is more stable than the CM between the backbone of PAP and hydroxy terminal groups. Further analysis reveals that in the case of [[NH]_PAP_] and [[O]_C=O_], the free energy of the transition state (TS) between CM and SShM is positive, while the free energy of the TS between CM and SAM is negative for [[NH]_PAP_] and [[O]_OH_]. This indicates the presence of a very stable HB between the backbone of PAP and ester terminal groups.

In our investigation, we explored the cluster-formation-fraction *f*_cluster_^(αβ)^(*s*) using the additional criterion (|*W*(*r*_CM_)| > 2*k*_B_ *T*). In the absence of PAP (*P* = 0 wt %), no cluster formation was observed in _H_PI_H_, _ω_PI_α1_, _ω_PI_α3_, and _ω_PI_α5_ melt systems as the weak association between terminal groups led to (|*W*(*r*_CM_)| ≈ *k*_B_ *T*). However, in the presence of 1 wt % PAP, stable clusters with ester α terminal groups were facilitated due to the strong HB between the backbone of PAP and the carbonyl group of ester α terminal. For *P* = 0 wt %, strong interactions between terminal groups (|*W*(*r*_CM_)| > 2*k*_B_ *T*)­led to the formation of stable clusters of various sizes in _ω_PI_α2_, _ω_PI_α4_, and _ω_PI_α6_, and the fraction of these stable clusters became more evident in the presence of PAP. Notably, the cluster-formation-fractions of tetra- and pentamer-clusters were considerably higher in _ω_PI_α2_ than in _ω_PI_α4_ and _ω_PI_α6_. Additionally, PAP played a role in assisting the formation of large-sized clusters with hydroxy α terminal groups. The presence of size two to nine clusters (Figure 12) provided strong evidence for the existence of multiple PJPs between PAP and the hydroxy and ester-terminated polyisoprene chains. Consequently, PAP molecules exhibited strong association with α terminal groups of *cis*-1,4-polyisoprene chains, which aligns with findings from previous experimental research.^5^ The visualization of these junction points can be observed in Figure 16 and Videos S1, S2, S3, S4, S5, and S6, corresponding to _ω_PI_α1_ + P, _ω_PI_α2_ + P, _ω_PI_α3_ + P, _ω_PI_α4_ + P,_ω_PI_α5_ + P, and _ω_PI_α6_ + P melt systems, respectively. These snapshots and videos are created by using VMD software.^117^ Ultimately, the formation of multiple PJPs significantly reduced the motility of ester- and hydroxy-terminated polyisoprene chains.

**Figure 16:**
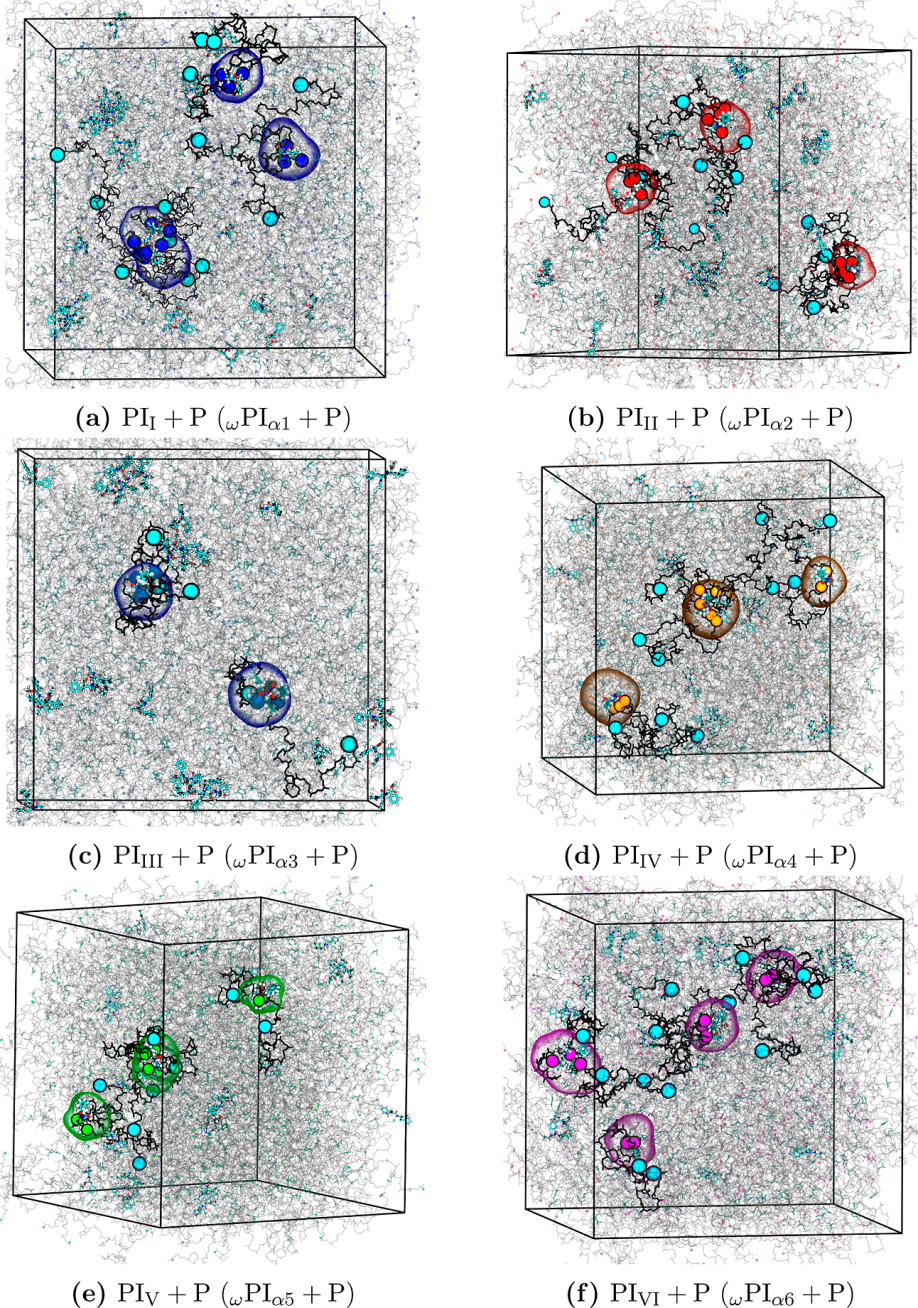
PJPs of [α1] around the dipeptide, [α2] around the dipeptide, [α3] around the dipeptide, [α4] around the dipeptide, [α5] around the dipeptide, and [α6] around the dipeptide in (a) PI_I_ + P, (b) PI_II_ + P, (c) PI_III_ + P, (d) PI_IV_ + P, (e) PI_V_ + P, and (f) PI_VI_ + P melt systems, respectively. The DMA group of ω terminals is represented by cyan color, while [α1], [α2], [α3], [α4], [α5], and [α6] terminals are depicted by blue, red, dark-blue, orange, green, and magenta colors, respectively. The backbone carbon and hydrogen atoms are shown in gray color.

In conclusion, our investigation focused on exploring the association of PAP with ω and α terminals, along with its impact on the dynamic behavior of polyisoprene chains in diverse melt systems. The observed weaker associations between [Phenyl] and [DMA] in comparison to [DMA] and [DMA] preclude the formation of stable clusters between PAP and ω terminals. The establishment of a robust hydrogen-bond network between the ester terminal groups and the PAP backbone validates the formation of stable physical junctions between PAP and ester terminal groups in _ω_PI_α1_ + P, _ω_PI_α3_ + P, and _ω_PI_α5_ + P. In contrast, the pronounced hydrogen-bond interactions involving [α2] and [α2], [PAP] and [α2], [α4] and [α4], [PAP] and [α4] and [α6] and [α6], [PAP] and [α6] facilitate the formation of large, stable clusters (*s* > 2). These observations provide strong evidence for the occurrence of multiple physical cross-linked points between hydroxy-terminated polyisoprene chains and PAP with hydroxy [α] terminal groups. The size of the physical junction among ester-terminals with PAP as a binder is not large, i.e., *s* ≈ 2. Our investigation reveals that the PJP between PAP and ester terminal groups exhibits enhanced stability compared to that between PAP and hydroxy terminal groups, a finding supported by the analysis of continuous and intermittent HB lifetimes. Furthermore, we observe the globular physical junction formed between ester terminal groups and PAP. Based on these conclusive outcomes, our results unequivocally reject the hypothesis of PAP (and proteins) associating with the ω-terminal. Nevertheless, it is important to acknowledge that our assumption regarding the chemical structure of the ω-terminal is derived from the work by Oouchi et al.,^1^ where the ω-terminal is considered as dimethyl allyl-(*trans*-1,4-isoprene)_2_. Due to the inherent difficulty in experimentally determining the chemical structure of *cis*-1,4 polyisoprene end-groups obtained from a rubber tree, the existence of an alternative chemical structure for the ω-terminal cannot be ruled out.

Our investigations have successfully verified the existence of hydrogen-bonded polar aggregates formed at the [α] terminals of polyisoprene chains in conjunction with PAP, a finding that aligns with earlier experimental research.^5^ These polar PJPs hold considerable potential as contributors to the intriguing phenomenon of strain-induced crystallization observed in NR. To further elucidate the underlying mechanisms, we plan to conduct comprehensive all-atom MD simulations of polyisoprene melt systems enriched with lipids. Through this forthcoming research, we aim to gain insights into how lipids influence the formation of PJPs involving both [α] and [ω] terminal groups of PI chains. We hypothesize that these junction points could play pivotal roles in endowing NR with its exceptional properties, such as high resistance to crack growth and the intriguing strain-induced crystallization behavior. Our future endeavors in this direction are expected to provide valuable knowledge for the advancement of rubber materials with enhanced performance characteristics.

## Supplementary Material















## Notes

Equilibrated structures of polymer melt systems; end-to-end distance *R*_ee_ and radius of gyration *R*_g_, mean square internal distances, end-to-end vector autocorrelation functions *C*(*t*), single-chain total dipole moment autocorrelation function ϕ_total_(*t*), time autocorrelation function of normal modes, RDFs, fitted survival probabilities *P*(τ), and intermittent HB correlation function *C*_HB_^I^(*t*) (PDF)
Formation of a PJP between [α1] terminal groups and PAP in the _ω_PI_α1_ + P melt system (MP4)
Formation of a PJP between [α2] terminal groups and PAP in the _ω_PI_α2_ + P melt system (MP4)
Formation of a PJP between [α3] terminal groups and PAP in the _ω_PI_α3_ + P melt system (MP4)
Formation of a PJP between [α4] terminal groups and PAP in the _ω_PI_α4_ + P melt system (MP4)
Formation of a PJP between [α5] terminal groups and PAP in the _ω_PI_α5_ + P melt system (MP4)
Formation of a PJP between [α6] terminal groups and PAP in the _ω_PI_α6_ + P melt system (MP4)
