# Supplementary material for: Effect of Impurities on the Formation of End-Group Clusters in Natural Rubber: Phenylalanine Dipeptide as an Impurity Protein
Source: Macromolecules. 2024 Mar 5;57(6):2588–608. doi: 10.1021/acs.macromol.3c01833 (PMC12728983; doi:10.1021/acs.macromol.3c01833)
Supplement: Supplementary file 1 [file ma3c01833_si_001.pdf]

Supporting Information: Effect of impurity in the formation of end-group clusters in natural rubber: Phenylalanine dipeptide as an impurity protein

Mayank Dixit* and Takashi Taniguchi*

Graduate School of Engineering, Kyoto University, Nishikyo-ku, Kyoto 615-8510, Japan

E-mail: dixit@cheme.kyoto-u.ac.jp; taniguch@cheme.kyoto-u.ac.jp

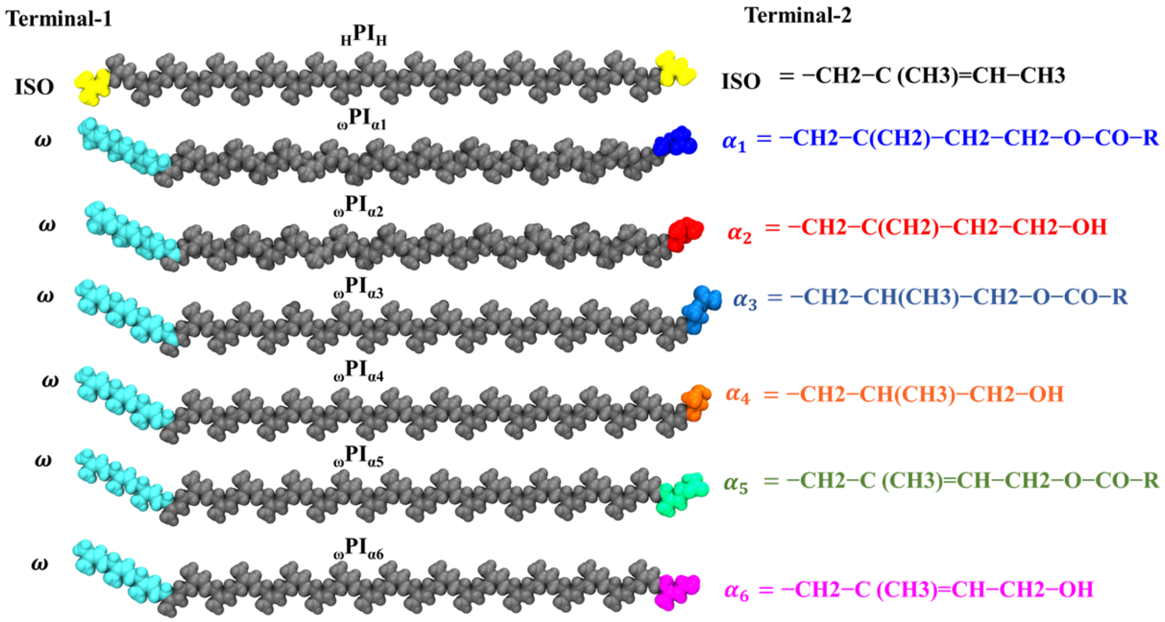

Figure S1: The extended structure of polymer chain of pure PI terminated by H (${}_{\mathrm{H}}\mathrm{PI}_{\mathrm{H}}$), ${}_{\omega}\mathrm{PI}_{\alpha 1}$, ${}_{\omega}\mathrm{PI}_{\alpha 2}$, ${}_{\omega}\mathrm{PI}_{\alpha 3}$, ${}_{\omega}\mathrm{PI}_{\alpha 4}$, ${}_{\omega}\mathrm{PI}_{\alpha 5}$ and ${}_{\omega}\mathrm{PI}_{\alpha 6}$ are shown. The end-isoprene residues of pure PI are shown by yellow color. ω-terminals (Dimethyl allyl group and two trans-1,4-isoprene) are shown by cyan color and $\alpha 1$, $\alpha 2$, $\alpha 3$, $\alpha 4$, $\alpha 5$ and $\alpha 6$ terminals are shown by blue, red, web-blue, orange, dark-green and magenta colors respectively. Backbone carbon and hydrogen atoms are shown by gray color.

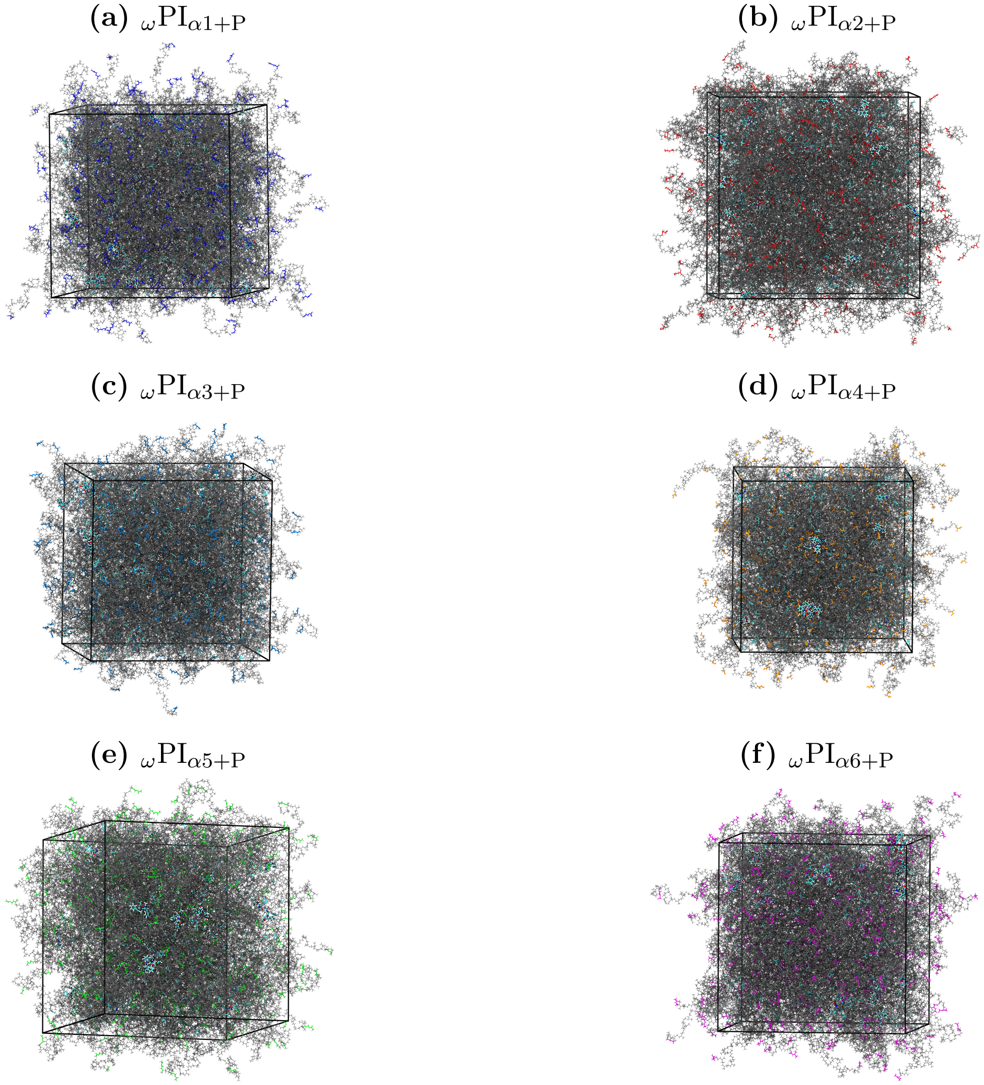

Figure S2: The equilibrated structures of (a) $_{\omega}\mathrm{PI}_{\alpha1+\mathrm{P}}$, (b) $_{\omega}\mathrm{PI}_{\alpha2+\mathrm{P}}$, (c) $_{\omega}\mathrm{PI}_{\alpha3+\mathrm{P}}$, (d) $_{\omega}\mathrm{PI}_{\alpha4+\mathrm{P}}$, (e) $_{\omega}\mathrm{PI}_{\alpha5+\mathrm{P}}$ and (f) $_{\omega}\mathrm{PI}_{\alpha6+\mathrm{P}}$ melt systems. Dimethyl allyl group of ω terminals (Dimethyl allyl group and two trans-1,4-isoprene) are shown by cyan color and $\alpha1$, $\alpha2$, $\alpha3$, $\alpha4$, $\alpha5$ and $\alpha6$ terminals are shown by blue, red, web-blue, orange, green and magenta colors respectively. Backbone carbon and hydrogen atoms are shown by gray color. The carbond, nitrogen, oxygen and hydrogen atoms of backbone of phenylalanine dipeptide molecules are shown by cyan, blue, red and white colors respectively.

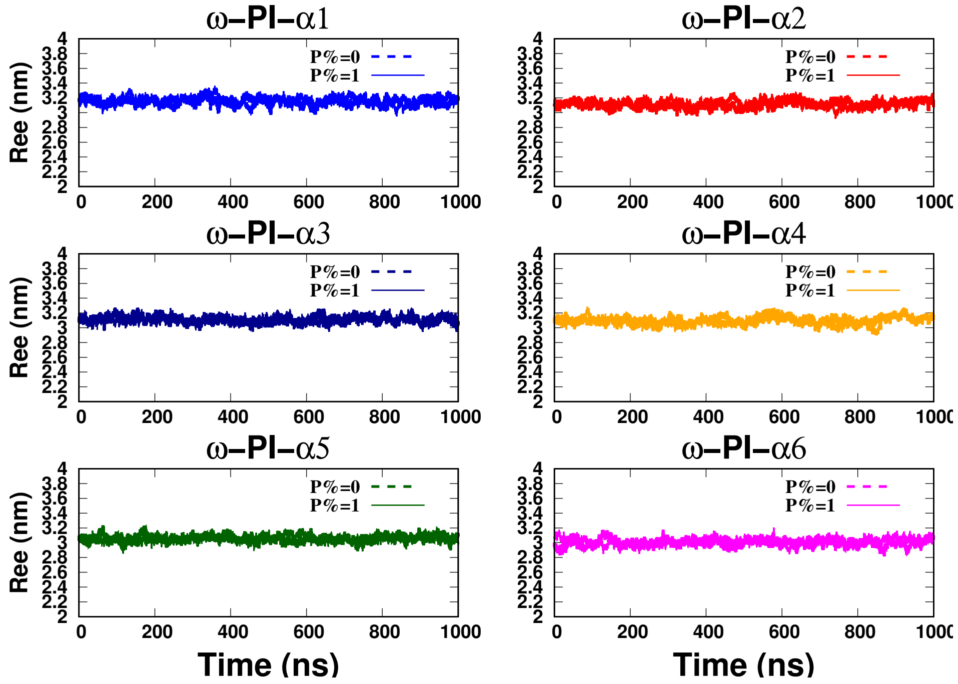

(a) The end-to-end distance vs time.

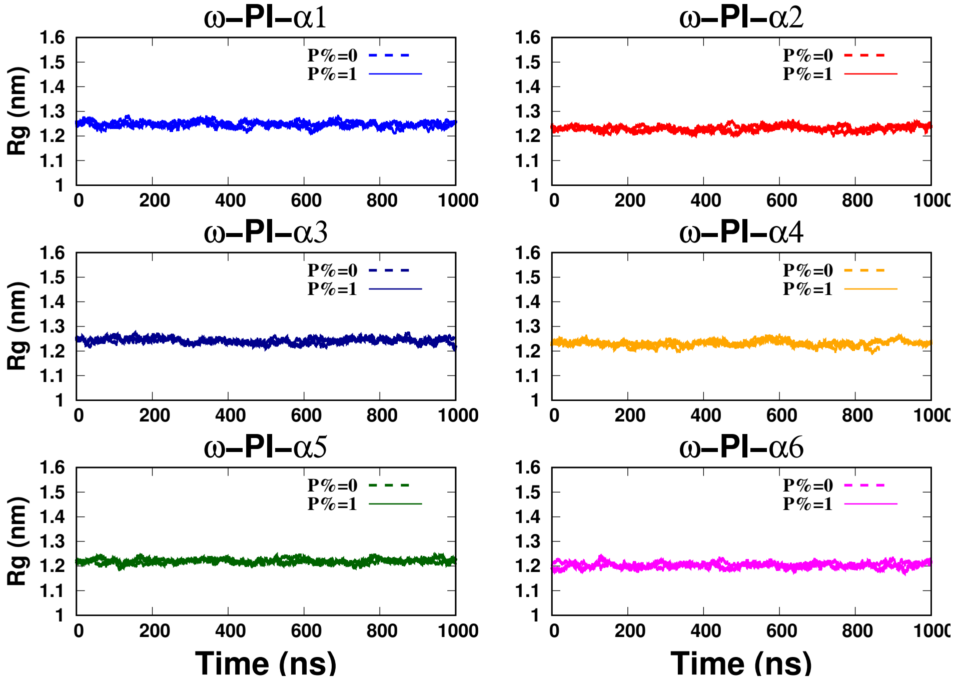

(b) The Radius of gyration vs time

Figure S3: The end-to-end distance vs time (a) and Radius of gyration vs time (b) for each melt system in NVT ensemble.

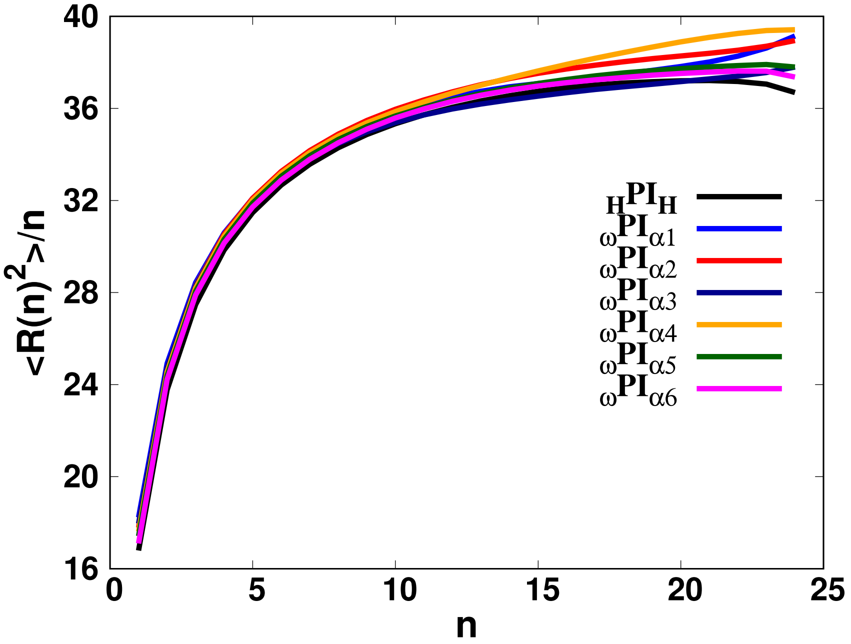

Figure S4: The mean square internal distances for the seven melt systems.

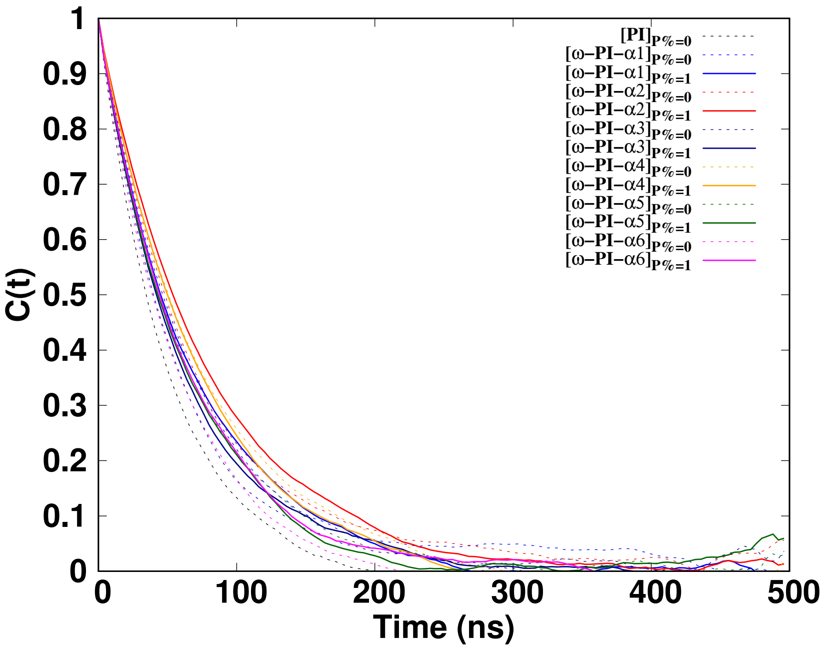

Figure S5: The average rotational time correlation function vs time for each melt system in NVT ensemble. The end-to-end vector auto correlation functions $C(t)$ is fitted into the Kohlrausch-Williams-Watts stretched exponential function to compute average relaxation time τ and β.

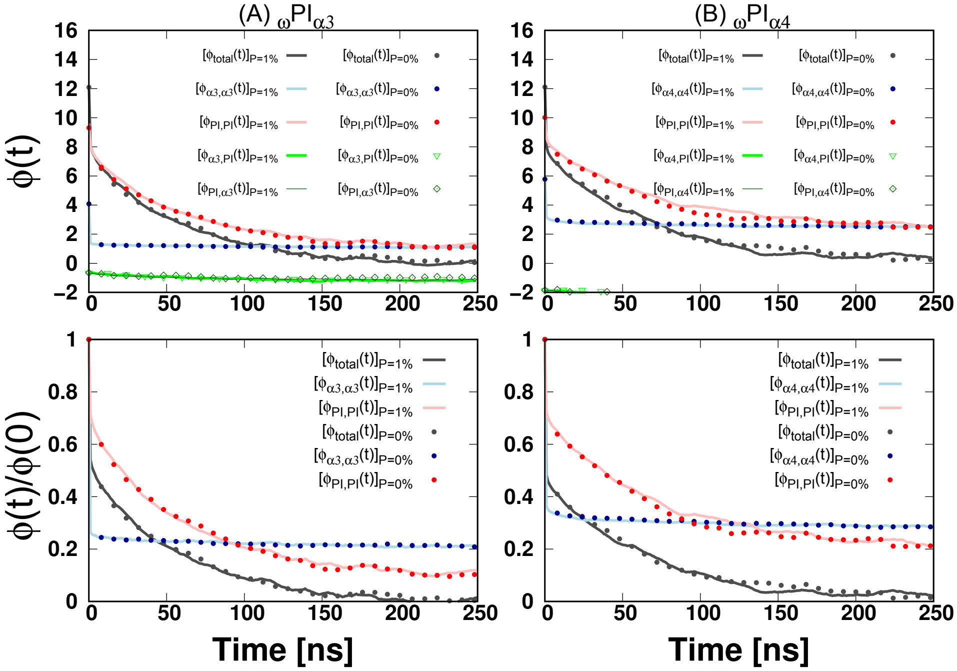

Figure S6: Single chain dipole moment autocorrelation functions vs time plot for $\mathrm{PI}_{\mathrm{III,IV}}$ and $\mathrm{PI}_{\mathrm{III,IV}} + \mathrm{P}$ melt systems. The decomposition of the single-chain total dipole moment autocorrelation function, denoted as $\phi_{\mathrm{total}}(t)$, involves a partition into four distinct components: $\phi_{\mathrm{PI,PI}}(t)$,$\phi_{\alpha n,\alpha n}(t)$, $\phi_{\mathrm{PI},\alpha n}(t)$, $\phi_{\alpha n,\mathrm{PI}}(t)$.

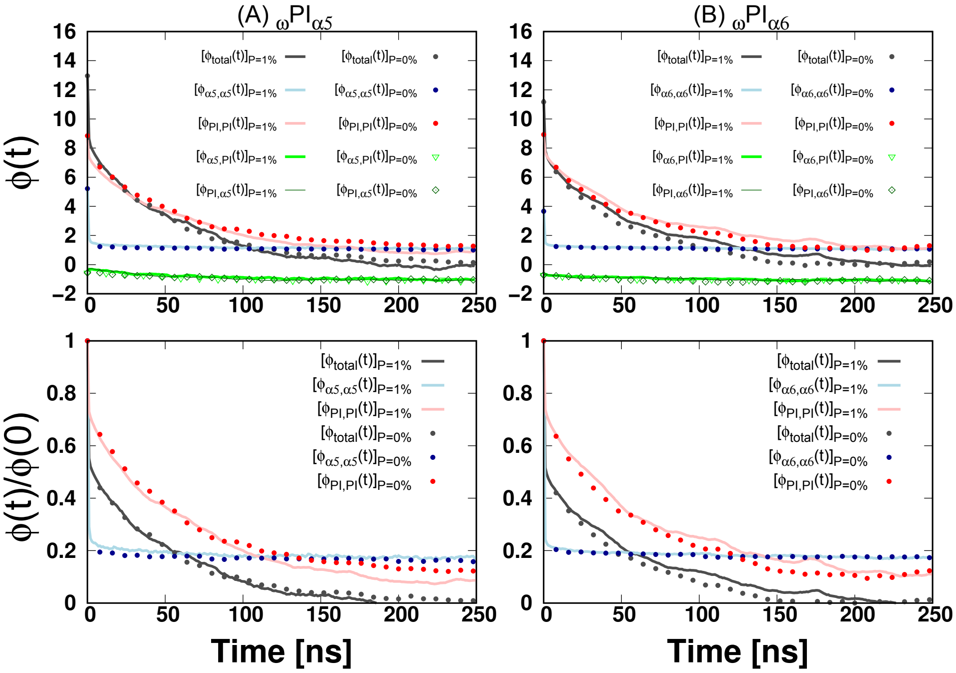

Figure S7: Single chain dipole moment autocorrelation functions vs time plot for for $\mathrm{PI_{V,VI}}$ and $\mathrm{PI_{V,VI}+P}$ melt systems. The decomposition of the single-chain total dipole moment autocorrelation function, denoted as $\phi_{\text{total}}(t)$, involves a partition into four distinct components: $\phi_{\text{PI,PI}}(t)$,$\phi_{\alpha n,\alpha n}(t)$, $\phi_{\text{PI},\alpha n}(t)$, $\phi_{\alpha n,\text{PI}}(t)$.

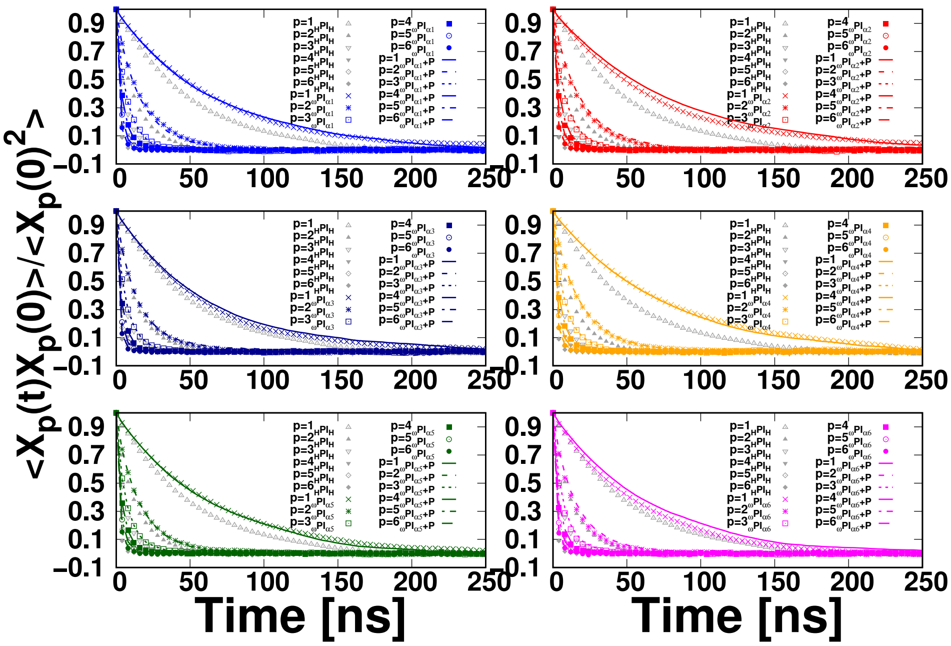

Figure S8: Time autocorrelation function of normal modes p =1, 2, 3, 4, 5 and 6 for each melt system, as obtained from the all-atom MD simulations.

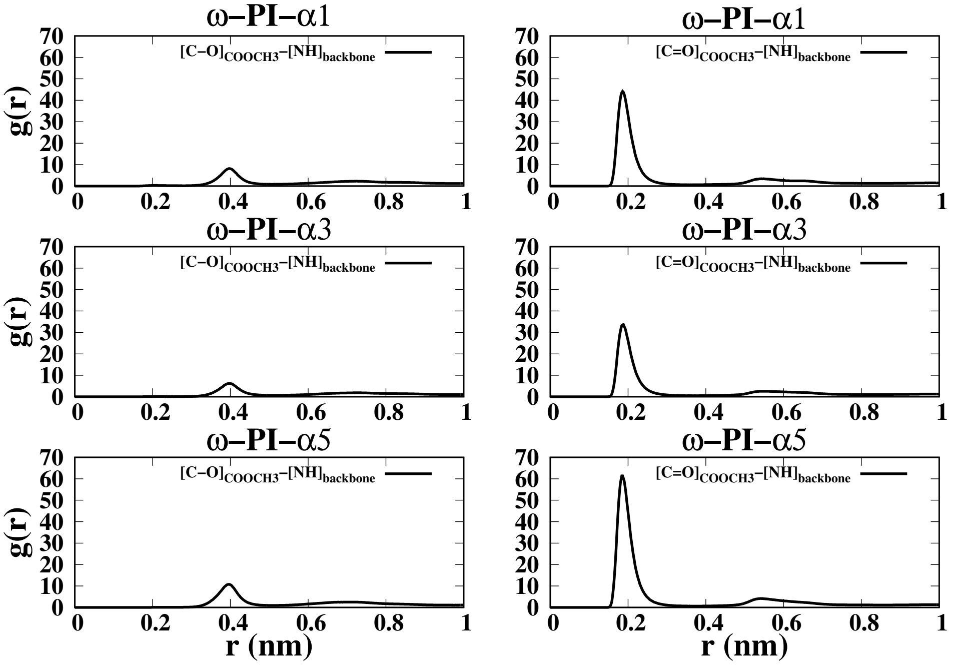

Figure S9: The radial distribution function of ester α terminals around the backbone of phenyl alanine dipeptide.

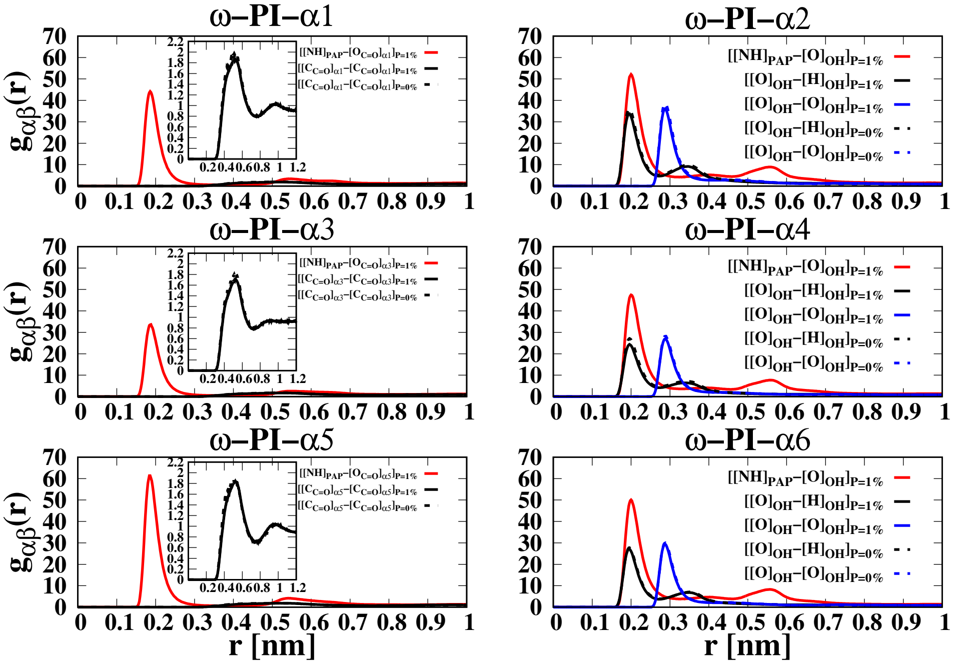

Figure S10: The radial distribution functions of the terminals around terminals and terminals around dipeptide molecules in the twelve types of cis polyisoprene systems. ″[Phenyl]″ stands for the center of mass of phenyl residue of phenylalanine dipeptide molecule. ″[DMA]″ stands for the center of mass of dimethyl allyl residue of ω terminal. ″$[C_{C=O}]$″ is expressing the carbon atom in the ester group in $\alpha 1$, $\alpha 3$ and $\alpha 5$-terminals. [O] and [H] stand for oxygen and hydrogen atom of the hydroxy group in $\alpha 2$, $\alpha 4$ and $\alpha 6$-terminals. As α and β in $g_{\alpha\beta}$, $[O_{C=O}]$,$[C_{C=O}]$, [Phenyl], [DMA], [O], [H] are considered. The RDF of β around α and (symbolically, expressed by $\alpha-\beta$) are drawn by the following colors : black for $([C_{C=O}]-[C_{C=O}])$, red for $[NH_{PAP}] - [O_{C=O}])$, black for $([O]_{OH} - [H]_{OH})$ and blue for $([O]_{OH} - [O]_{OH})$ and red for $[NH_{PAP}] - [O_{OH}])$. Solid lines are correspoding to $P = 1$wt% and dashed lines are for $P = 0$wt%.

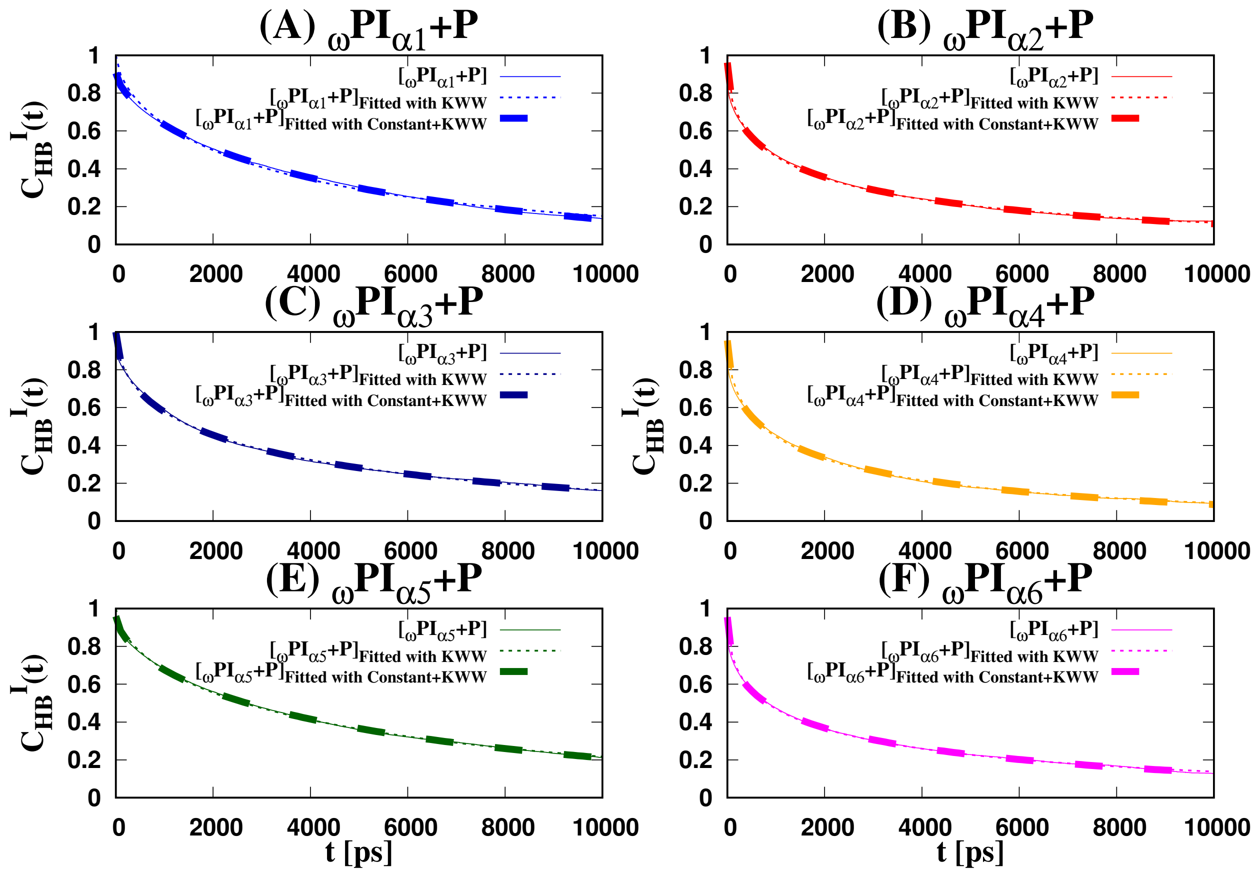

Figure S11: Intermittent $C_{\mathrm{HB}}^{\mathrm{I}}(t)$ hydrogen bond correlation functions for $_{\omega}\mathrm{PI}_{\alpha 1}+\mathrm{P}$ (A), $_{\omega}\mathrm{PI}_{\alpha 2}+\mathrm{P}$(B), $_{\omega}\mathrm{PI}_{\alpha 3}+\mathrm{P}$(C), $_{\omega}\mathrm{PI}_{\alpha 4}+\mathrm{P}$(D), $_{\omega}\mathrm{PI}_{\alpha 5}+\mathrm{P}$(E) and $_{\omega}\mathrm{PI}_{\alpha 6}+\mathrm{P}$(F). $C_{\mathrm{HB}}^{\mathrm{I}}(t)$ are shown by solid lines. We also fitted intermittent hydrogen bond correlation function into two types of Kohlrausch-Williams-Watts (KWW) stretched exponential functions i.e. $C_{\mathrm{HB}}^{\prime\mathrm{I}}(t) = C_{\mathrm{HB}}^{\prime\mathrm{I}}(0)\exp[-(t/\tau_{\mathrm{HB}}^{\prime\mathrm{I}})^{\beta_{\mathrm{HB}}^{\prime\mathrm{I}}}]$ and $C_{\mathrm{HB}}^{\mathrm{I}}(t) = P_{\mathrm{HB}}^{\mathrm{I}} + C_{\mathrm{HB}}^{\mathrm{I}}(0)\exp[-(t/\tau_{\mathrm{HB}}^{\mathrm{I}})^{\beta_{\mathrm{HB}}^{\mathrm{I}}}]$. The fitted curves of $C_{\mathrm{HB}}^{\prime\mathrm{I}}(t)$ and $C_{\mathrm{HB}}^{\mathrm{I}}(t)$ are shown by thin-dashed and thick dashed lines respectively.

Table S1: The rotational relaxation time $\tau_{\mathrm{rot.}}$ and the stretching exponent β. The rotational correlation function $C(t)$ is fitted using the Kohlrausch-Williams-Watts stretched exponential function to determine the rotational relaxation time $\tau_{\mathrm{rot.}}$ and the stretching exponent β.

Systems	Type of alpha terminal	Average relaxation time $\tau_{\mathrm{rot.}}$/[ns]		Stretching exponent β	
		P = 0wt%	P = 1wt%	P = 0wt%	P = 1wt%
$_{\mathrm{H}}\mathrm{PI}_{\mathrm{H}}$		42.50±0.01	-	0.88	-
$_{\omega}\mathrm{PI}_{\alpha 1}$	Ester	53.74±0.01	55.39±0.01	0.82	0.86
$_{\omega}\mathrm{PI}_{\alpha 3}$		47.73±0.01	50.51±0.01	0.88	0.87
$_{\omega}\mathrm{PI}_{\alpha 5}$		53.98±0.02	54.26±0.02	0.88	0.93
$_{\omega}\mathrm{PI}_{\alpha 2}$	Hydroxy	56.98±0.04	67.19±0.01	0.85	0.92
$_{\omega}\mathrm{PI}_{\alpha 4}$		61.12±0.03	62.33±0.01	0.86	0.92
$_{\omega}\mathrm{PI}_{\alpha 6}$		49.05±0.01	55.27±0.01	0.97	0.91

Table S2: The longest chain relaxation time τ_{R} (τ_1), providing a comprehensive perspective on the temporal evolution. The time autocorrelation function $\langle \mathbf{X}_p(t)\cdot\mathbf{X}_p(0)\rangle/\langle \mathbf{X}_p(0)\cdot\mathbf{X}_p(0)\rangle$ undergoes fitting with a single exponential function, serving as a crucial tool in extracting the chain relaxation time τ_p. The rotational correlation function $C(t)$ is fitted using the simple exponential decay function ($\beta = 1$) to determine the rotational relaxation time τ_{rot}.

Systems	Type of alpha terminal	τ_{R} (τ_1) [ns]		$\tau_{\mathrm{rot.}}$ [ns] ($\beta = 1$)		$\tau_{\mathrm{R}}/\tau_{\mathrm{rot.}}$	
		P = 0wt%	P = 1wt%	P = 0wt%	P = 1wt%	P = 0wt%	P = 1wt%
$_{\mathrm{H}}\mathrm{PI}_{\mathrm{H}}$		48.88±0.01	-	45.41±0.01	-	1.07	-
$_{\omega}\mathrm{PI}_{\alpha 1}$	Ester	66.99±0.02	68.04±0.01	57.04±0.02	62.70±0.01	1.17	1.08
$_{\omega}\mathrm{PI}_{\alpha 3}$		56.56±0.01	62.72±0.01	53.10±0.01	56.84±0.01	1.06	1.10
$_{\omega}\mathrm{PI}_{\alpha 5}$		64.83±0.02	63.74±0.02	56.79±0.02	57.53±0.02	1.14	1.10
$_{\omega}\mathrm{PI}_{\alpha 2}$	Hydroxy	72.55±0.04	79.24±0.01	65.71±0.03	71.90±0.01	1.10	1.10
$_{\omega}\mathrm{PI}_{\alpha 4}$		72.70±0.03	72.52±0.01	64.14±0.03	66.85±0.01	1.13	1.08
$_{\omega}\mathrm{PI}_{\alpha 6}$		57.07±0.01	64.44±0.01	57.23±0.01	64.78±0.01	0.99	0.99

Table S3: The hydrogen bond (HB) relaxation time $\tau_{\rm HB}$ and stretching exponent $\beta_{\rm HB}$ of $[{\rm NH}]_{\rm PAP} - [\alpha n]$ melt systems ($n = 1, 2, 3, 4, 5, 6$).

Systems	HB relaxation time ($\tau_{\rm HB}$)/[ps]		Stretching exponent ($\beta_{\rm HB}$)		Constant ($P_{\rm HB}^{\prime \rm I}$)	χ^2	
	($\tau_{\rm HB}^{\prime \rm I}$)	($\tau_{\rm HB}^{\rm I}$)	($\beta_{\rm HB}^{\prime \rm I}$)	($\beta_{\rm HB}^{\rm I}$)	($P_{\rm HB}^{\rm I}$)	($\chi_{\rm HB}^{\prime 2\rm I}$)	($\chi_{\rm HB}^{2\rm I}$)
$[{\rm NH}]_{\rm PAP}$-$[\alpha 1]$	3577.0±5.1	5502.9±12.1	0.6216±0.0012	0.6664±0.0005	0.0931±0.0005	20.4818×10^{-5}	1.4136×10^{-5}
$[{\rm NH}]_{\rm PAP}$-$[\alpha 2]$	1811.2±2.2	2337.4±16.1	0.4519±0.0005	0.4482±0.0004	0.0381±0.0010	5.07591×10^{-5}	3.13899×10^{-5}
$[{\rm NH}]_{\rm PAP}$-$[\alpha 3]$	3164.5±2.5	3141.1±19.3	0.5188±0.0005	0.5187±0.0005	0.0012±0.0010	4.20458×10^{-5}	4.20398×10^{-5}
$[{\rm NH}]_{\rm PAP}$-$[\alpha 4]$	1589.6±2.5	2133.4±16.2	0.4643±0.0006	0.4564±0.0005	0.0447±0.0011	7.6369×10^{-5}	4.46367×10^{-5}
$[{\rm NH}]_{\rm PAP}$-$[\alpha 5]$	4895.04±3.2	5908.53±15.53	0.5928±0.0005	0.6156±0.0006	0.0399±0.0006	4.25949×10^{-5}	1.46063×10^{-5}
$[{\rm NH}]_{\rm PAP}$-$[\alpha 6]$	1956.03±2.07	2683.78±15.88	0.4191±0.0004	0.4185±0.0002	0.0445±0.0008	3.51361×10^{-5}	1.55786×10^{-5}
